# Antibiotic-Induced Neutropenia in Pediatric Patients: New Insights From Pharmacoepidemiological Analyses and a Systematic Review

**DOI:** 10.3389/fphar.2022.877932

**Published:** 2022-06-02

**Authors:** Vera Battini, Alessandra Mari, Michele Gringeri, Francesca Casini, Francesco Bergamaschi, Giulia Mosini, Greta Guarnieri, Marco Pozzi, Maria Nobile, Gianvincenzo Zuccotti, Emilio Clementi, Sonia Radice, Valentina Fabiano, Carla Carnovale

**Affiliations:** ^1^ Unit of Clinical Pharmacology, Department of Biomedical and Clinical Sciences, “Luigi Sacco” University Hospital, Università degli Studi di Milano, Milan, Italy; ^2^ Unit of Pediatrics, Department of Biomedical and Clinical Sciences, “Vittore Buzzi” Children’s University Hospital, Università degli Studi di Milano, Milan, Italy; ^3^ Scientific Institute IRCCS E. Medea, Bosisio Parini, Lecco, Italy

**Keywords:** neutropenia, antibiotics, pediatrics, pharmacovigilance, FAERS

## Abstract

**Aim:** to characterize pediatric cases of antibiotic-associated neutropenia through a multidisciplinary approach, focusing on the temporal association between the wide spectrum of treatment options and the occurrence of this relatively uncommon but potentially clinically relevant adverse event.

**Methods:** we carried out a pharmacoepidemiological analysis based on the FDA Adverse Event Reporting System (FAERS) database, a retrospective chart review and a systematic review of the literature, focusing on the time to onset (TTO) of this side effect, in the pediatric clinical setting.

**Results:** A total of 281 antibiotic-related neutropenia events, involving 11 categories of antibiotics, were included in the time to onset analysis. The median TTO ranged from 4 to 60 days after the start of the therapy. A shorter median TTO was found from the retrospective chart review [16 patients: median days (25th-75th percentiles) = 4 (3–5)], compared to 15 (9–18) vs. 10 (6–18) for literature (224 patients) and FAERS (41 cases), respectively. The Anatomical Therapeutic Chemical classes, J01X, J01F, J01E and J04A, and the median TTOs retrieved from more than one source revealed high accordance (*p* > 0.05), with J01X causing neutropenia in less than a week and J01F/J01E/J04A in more than 10 days. Antibiotics were discontinued in nearly 34% of cases. In FDA Adverse Event Reporting System reports, half of the patients experiencing neutropenia were hospitalized.

**Conclusion:** Whereas antibiotic associated neutropenia is benign in the majority of cases, yet it should not be neglected as, even if rarely, it may put children at higher risk of clinical consequences. Clinicians’ awareness of antibiotic-associated neutropenia and its mode of presentation contributes to the continuous process of monitoring safety of antibiotics.

## 1 Introduction

Drug-induced neutropenia is a relatively rare but potentially fatal disorder (with a significant impact on health care costs); it occurs in susceptible individuals, with an incidence of 2.4–15.4 cases per million population (Strom et al., 1992). It also raises serious concern during the development of new drugs (Uetrecht, 2007) because it can be missed in clinical trials due to its low incidence. This issue is of paramount importance since, in severe cases, it can even lead to withdrawal of a drug from the market (Thompson et al., 2016).

Neutrophils play a crucial role in the defence and control of infections, especially bacterial ones; the normal range for the absolute neutrophil count (ANC) varies with age; the lower limit of normal is 5,000/ml (5.0 × 109/L) for the first week of life, then falls to 1,000/ml (1.0 × 109/L) between 2 weeks and 1 year of age (Bhatt and Saleem, 2004; Hsieh et al., 2007; Segel and Halterman, 2008; Newburger and Dale, 2013; Barg et al., 2015; Dale, 2017). According to the ANC, neutropenia can be graded as mild (1,000–1,500 cells/μL), moderate (1,000–1,500 cells/μL) and severe (<500 cells/μL) (Bhatt and Saleem, 2004).

Over the last 20 years, a number of medications have been strongly implicated as potential causes of idiosyncratic neutropenia, including antithyroid agents, psychotropic drugs, anticonvulsants, and antibiotics (Andersohn et al., 2007a; Andrès et al., 2011; Johnston and Uetrecht, 2015; Andrès et al., 2017).

Antibiotics are the most commonly prescribed therapy to children (Nicolini et al., 2014) and, especially for preterm infants or children in neurological rehabilitation, who are more prone to the risk of bacterial infections (Bhutta and Black, 2013; Sankar et al., 2016; Shane et al., 2017), antibiotics are an essential lifesaving treatment. The use of unlicensed or off-label antibiotics is indeed a common practice in neonatal intensive care units (Kumar et al., 2008).

Up to 30% of children exposed to long-term antibiotic therapy (>14 days) is expected to develop adverse effects, with an incidence of neutropenia that varies dramatically in publications (Gomez et al., 2001; Maraqa et al., 2002; Andersohn et al., 2007b; Olson et al., 2015; Tribble et al., 2020).

Drug-induced neutropenia is the second commonest cause of acquired acute neutropenia in children, after post-infectious neutropenia (Barg et al., 2015; Knight, 2015). The true incidence of this phenomenon is not known, as most reports focus on the rare and more severe agranulocytosis, which has an incidence of 1–10 cases per million per year.

Clinicians who prescribe to and treat patients with antibiotics regularly (Andersohn et al., 2007a; Andrès et al., 2011, 2017) and are aware of antibiotic side effects, may not detect neutropenia since patients developing this rare side effect are either asymptomatic or experience non-specific symptoms such as fever and skin rash (Andersohn et al., 2007a; Andrès et al., 2011, 2017). Moreover, in the context of septicemia, severe sepsis or viral infections, the role of antibiotics as causative drugs of neutropenia is often difficult to define (Andrès et al., 2017).

Affected patients experience severe neutropenia within several weeks to several months after first exposure to a drug; the temporal association between the wide spectrum of treatment options and the occurrence of neutropenia has not been clarified yet, negatively impacting on its early identification and eventual management.

The pathogenesis of antibiotic-induced neutropenia is complex, not fully understood yet and potentially multifactorial (Murphy et al., 1985; Olaison et al., 1999; Andrès et al., 2017). Some studies have suggested an immune-mediated etiology due to production of anti-neutrophil antibodies, similarly to the phenomenon of penicillin-induced hemolytic anemia (Kirkwood et al., 1983; Murphy et al., 1983, 1985; Salama et al., 1989).

Other reports have described a myelosuppressive effect of antibiotics, by demonstrating a lack of differentiated myeloid elements in bone marrow aspirates of subjects with antibiotic-induced neutropenia (Neftel et al., 1985). Recent studies have also suggested that the fecal microbiota regulate the number of circulating neutrophils, and that microbiota changes observed in course of antibiotic therapy may be linked to antibiotic-induced neutropenia (Balmer et al., 2014; Zhang et al., 2015; Josefsdottir et al., 2017).

Although the issue of antibiotic-induced neutropenia in the general population has been previously addressed (Holz et al., 2021), a unique focus on the pediatric population is missing; it would provide a more defined and comprehensive framework on the topic, underlining specific problems and details, and contributing to fill the gap in knowledge, thus helping physicians to face it. To this end we decided to proceed through the gathering of data by using an integrated approach based on pharmacoepidemiological analyses and a systematic review of all the currently available pediatric evidence; this strategy allows to define specifically aspects not retrievable otherwise, namely time to onset (TTO), distribution of agent groups, duration and treatment options, contributing to increase awareness about this potentially dangerous side effect among clinicians, towards improving of its management.

Despite the intrinsic limitations, we used the spontaneous reporting systems FDA Adverse Event Reporting System (FAERS), as it still represent a valuable source of real-world data about the safety/efficacy profile of specific drugs; it also allows to compare therapeutic options, gain relevant insights on potential mechanisms of adverse drug reactions (ADRs**)** (Carnovale et al., 2018, 2019a, 2019b; Mazhar et al., 2019)**,** and estimate the time to onset of ADRs (Mazhar et al., 2021), thus contributing to prevent ADRs and improve the pharmacological management of iatrogenic disorders. The results we obtained provide new insights towards improving the diagnosis of antibiotic-induced neutropenia, in the pediatric clinical setting.

## 2 Patients and Methods

### 2.1 Pharmacovigilance Study

Data were obtained from the FAERS, one of the largest and most comprehensive spontaneous reporting system databases. It receives millions of reports of adverse events per year from healthcare practitioners, consumers, companies, and other sources, concerning drugs. Adverse events are recorded in the FAERS using the Medical Dictionary for Regulatory Activities (MedDRA^®^) preferred terms. The database is largely used to detect novel drug-related safety events, to identify possible mechanisms of adverse events, to explore potential drug-drug interactions (Carnovale et al., 2019b; Mazhar et al., 2019). Adverse events recorded in the FAERS were downloaded from the Food and Drug Administration (FDA) website (FDA Adverse Event Reporting System, 2019 (FAERS) Quarterly Data Extract Files). The database consists of seven datasets, namely patient demographic and administrative information (file descriptor DEMO), drug and biologic information (DRUG), adverse events (REAC), patient outcomes (OUTC), report sources (RPSR), start and end dates of drug therapy (THER), and indications for use/diagnosis (INDI). These seven datasets were joined by unique identification numbers for each FAERS report and a relational database was built. Data extraction was restricted to reports without missing values for age and gender. Duplicate records were detected and deleted accordingly as previously described (Carnovale et al., 2018).

The cohort was retrieved from the FAERS database in the period covering the first quarter of 2010 to the second quarter of 2021 and consisted of all adverse events (AEs) occurred in paediatric patients (<18 years.o.). Since neutropenia must be carefully diagnosed in patients under the age of 18, we limit data extraction to those Individual Case Safety Reports (ICSRs) reported by physicians. A custom list of neutropenia-related event terms was then *ad hoc* created, combining different Preferred Terms that contain a range of Lowest Level Terms (LLTs) reflecting the same medical concept expressed by synonyms and lexical variants (Fescharek et al., 2004). After a review of all LLTs in MedDRA, 11 terms were selected: band neutrophil count decreased, band neutrophil percentage decreased, granulocyte count decreased, granulocyte percentage decreased, granulocytes maturation arrest, granulocytopenia, idiopathic neutropenia, neutropenia, neutrophil count decreased, neutrophil count abnormal, neutrophil percentage decreased. We filtered for ICSRs reporting at least one of the LLT above mentioned. At the same time, we excluded those ICSRs where the terms “sepsis” and “myelosuppression” were reported concomitantly with neutropenia. Finally, we included only those cases involving at least one antibiotic [Anatomical Therapeutic Chemical (ATC) code: J01] reported as “suspect drug” and clearly specifying in the report the start date of the therapy. If a patient had been treated with more than one antibiotic and the information was available for all the antibiotics, the single ICSR was split in more cases.

### 2.2 Case Series

We retrospectively reviewed medical records of children treated with oral (PO) or intravenous (IV) antibiotic therapy for bacterial infections, who presented neutropenia in the course of treatment. We considered patients who received inpatient antibiotic therapy in our Pediatric Department, Vittore Buzzi University Children’s Hospital, Milan, from 1 January 2020, until 30 June 2021.

To avoid confounding factors regarding neutropenia’s etiology, we included in the study only patients who presented neutropenia after the introduction of the antibiotic therapy, considering the pivotal role of the temporal relationship to assess causality. We have excluded patients with other possible underlying causes of neutropenia, including oncologic diseases, concomitant viral infections, bacterial sepsis, personal history of autoimmune disease, cyclic neutropenia or any pre-existent neutropenia. Neonates were not included in our analysis.

Furthermore, we assessed improvement of the neutrophil count over time in all patients.

Patients were included if complete blood count (CBC) had been performed at time of diagnosis (T0), before starting antibiotic therapy, and if at least one other CBC analysis was made for clinical necessity after starting therapy.

For the selected patients, we collected data on neutropenia’s TTO (i.e., the number of days between the beginning of antibiotic therapy and the detection of neutropenia) analyzing changes in CBC. Neutropenia was defined as an absolute neutrophil count (ANC) lower than 1,500/mmc (Bhatt and Saleem, 2004; Segel and Halterman, 2008).

### 2.3 Systematic Review

We performed a systematic review in accordance with the Preferred Reporting Items for Systematic Reviews and Meta-Analyses (PRISMA) guidelines (Page et al., 2021). We searched PubMed/MEDLINE, Embase, and the ClinicalTrials.gov database up to 21 January 2021 for the evidence of neutropenia following antibiotic treatment in a paediatric setting. The complete PubMed search string is described in the Supplementary Material S1. The search strategy was adapted as needed for each database. Essentially, we used the following terms combined with the Boolean operator “AND”: antibiotic therapy, neutropenia and adverse drug reaction, in order to obtain a wide selection of articles to be subsequently screened individually.

#### 2.3.1 Eligibility Criteria

Inclusion criteria were the following: any clinical trial, cohort or case-control study, case-report or case-series that reported a decrease in the neutrophil count that was ascribed to the treatment with one or a combination of antibiotics in at least one paediatric patient (neonates were excluded because of rapidly changing normal values references in the first weeks of life and consequently variable definitions of neutropenia at this age). We did not contact authors for unpublished data.

The formal assessment of causal relationship between neutropenia and antibacterial drugs was not included in the list of inclusion criteria. However, in order to avoid confounding factors, studies were also excluded if neutropenia occurred in patients affected by conditions that could trigger it, such as some specific infections (e.g., *salmonella typhi*, beta-haemolytic *streptococcus*, brucellosis, aspergillosis, malaria) and some concomitant diseases (e.g., oncologic and hematologic diseases). Studies reporting patients under concomitant therapy (e.g., chemotherapy, post-transplant anti-rejection medication) that could concur in the development of neutropenia were also discarded.

Reviews, systematic reviews, meta-analyses, guidelines, book chapters, unpublished theses, and *in vitro*/animal study were excluded, as well as articles written in languages other than English, French or Spanish. Moreover, any study referred to adult patients were excluded, as well as those where data concerning the paediatric population was not discernible from the adult one and studies in which antibiotics administration did not imply a systemic distribution (i.e., topic administration).

#### 2.3.2 Study Selection

After duplicate removal, our search results were screened by title and abstract and those potentially relevant were retrieved in full text and assessed for eligibility based on our prespecified inclusion criteria. The entire search process was performed by two independent researchers and disagreements about eligibility were solved upon reaching a consensus with a third investigator.

#### 2.3.3 Outcome Measures

Primary outcome was change (from baseline) in neutrophil count (N/mmc) associated with antibiotic use. Studies that reported only general leukopenia, or pancytopenia, were disregarded (and categorized as “no outcome of interest”), as were studies that did not specify the administered antibiotic, generically referring to “antibiotic therapy” (categorized as “no drug of interest”).

#### 2.3.4 Data Extraction

For each included study, we extracted the following information: study design (study type, study duration, sample size), patient characteristics (age, sex, and number exposed to antibiotics), therapy (drug name, dose, route of administration, reason for use, duration of treatment, and concomitant medication) and outcomes (number of cases out of exposed, TTO, neutropenia laboratory values, symptoms and complications, antibiotic discontinuation, days to resolution).

In view of the nature of our aim (i.e., collecting ADRs as they were reported in the eligible studies) we did not perform a formal assess of the quality of studies. However, to properly discuss findings we took into account study design and methodological aspects of eligible studies included in the systematic review.

### 2.4 Time-to-Onset Analysis

In the observational cohort, TTO was first calculated for each patient from the start of the therapy to the date of the laboratory data confirming neutropenia.

As for the systematic review, TTO was measured using the start date of the treatment and the date of neutropenia occurrence, by retrieving specific information provided by authors. Whether not explicitly reported, it was estimated using the median duration of treatment.

From the FAERS database, TTO was calculated from the time of the patient’s start date of the treatment (as reported in the ICSRs) to the occurrence of the neutropenia.

Drugs were merged following the Anatomical Therapeutic Chemical (ATC) codes and the median TTO was then calculated.

### 2.5 Statistical Analyses

Descriptive analysis was performed in terms of age, sex and the use of concomitant medications. Reduced levels in WBC and neutrophils were tested with one-tailed Wilcoxon test. The median (25th–75th percentiles) duration was used to evaluate the TTO. In order to compare our results concerning TTO, the two-tailed Mann-Whtiney test was used between two groups and two-tailed ANOVA Kruskall-Wallis among three groups. Concerning all tests, significance was set at a *p*-value of 0.05. Data reading, filtering, processing and statistical analysis were performed through RStudio (R Core Team, 2019).

## 3 Results

### 3.1 Pharmacovigilance Study

From the 63,084 pediatric ICSRs sent by physicians to the FAERS, 1,210 (1.91%) reported neutropenia as ADR; of these, 26 (2.14%) reported antibiotics as “suspect drugs” involved in the occurrence of neutropenia and provided data both on start therapy and AE dates. Details of all the retrieved cases related to the occurrence of Antibiotic-associated neutropenia from the FAERS are reported in the Supplementary Table S1. The mean age of the patients was 10 ± 6 years (min-max: 0–17) and 54% (*n* = 14) were females. Indications were largely heterogenous, but all related to serious infection like meningitis (*n* = 2), pyelonephritis (*n* = 1), osteomyelitis (*n* = 2). Concomitant medications were used in 25 (89%) patients and 24 (86%) used more than one antibiotic, mostly intravenous administered. 13 (50%) ICSRs required hospitalization; however, the remaining reports were related to other serious events, too (unfortunately, the reporter did not specify what type of other events). Only three ICSRs reported “neutropenia” as unique AE, while the other cases described the AE by using several MedDRA terms especially related to a systemic reaction against the suspect drugs. After splitting the ICSRs, 41 cases were available for the TTO analysis.

### 3.2 Case Series

Sixteen patients (9 females, 7 males) treated with intravenous (IV) or oral (PO) antibiotic therapy who developed neutropenia in course of treatment and did not present the above-mentioned exclusion criteria were included in our analysis. Patients’ age ranged from 1 to 51 months [median (25th–75th percentiles) = 5.5 (2.5–21)]; they were treated for pyelonephritis (*n* = 12, 75%), osteomyelitis (*n* = 2, 12.5%) and soft tissues infections (*n* = 2, 12.5%). All patients were previously healthy and did not receive any medications other than the prescribed antibiotics, except for paracetamol or ibuprofen for fever and pain relief.

**FIGURE 1:**
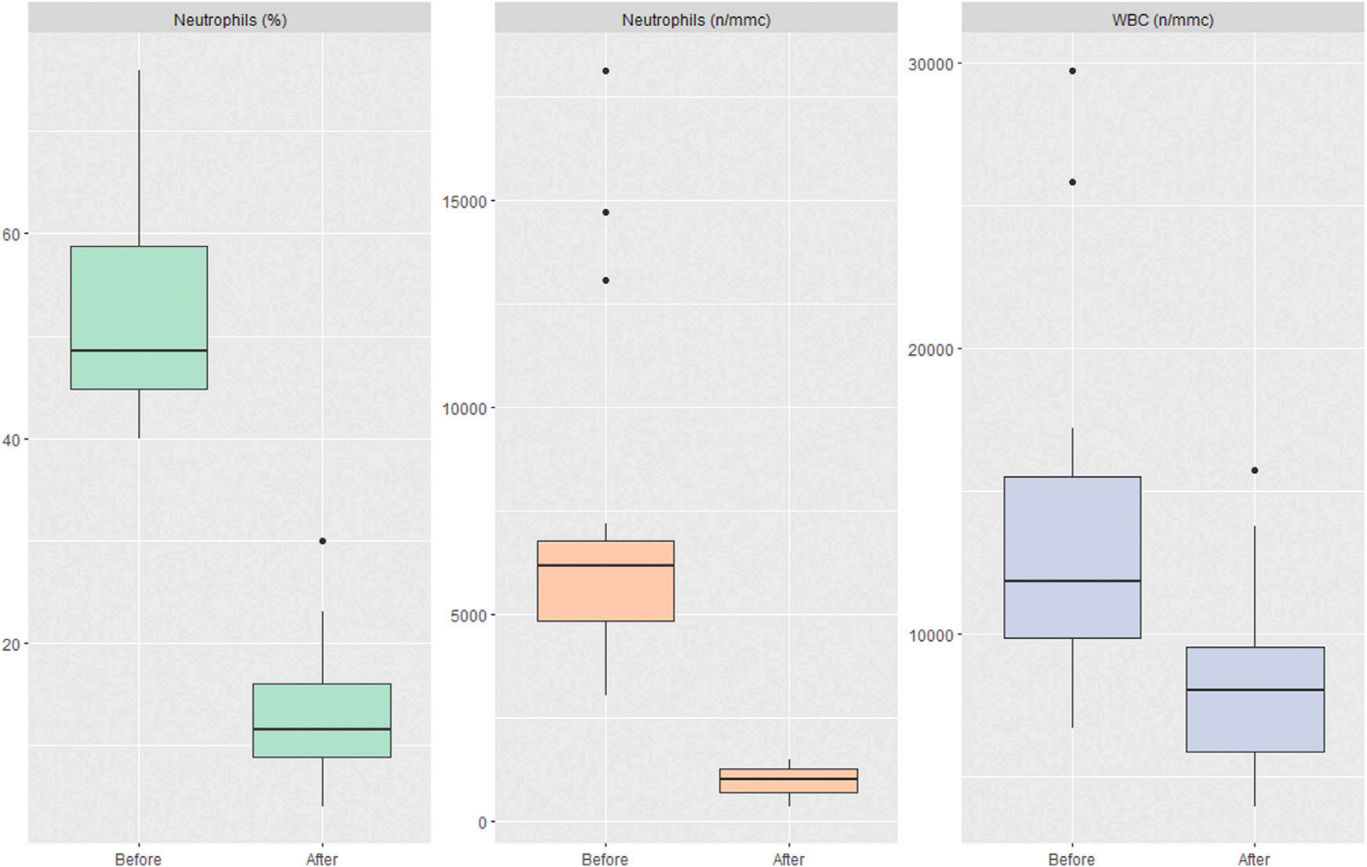
The median of the pre-and post-therapy neutrophils (%, n/mmc) and WBC levels (n/mmc).

IV ceftriaxone was the most frequently used antibiotic (*n* = 6), followed by PO amoxicillin/clavulanic acid (*n* = 4) and IV ampicillin/sulbactam (*n* = 2). The remaining four patients received respectively IV meropenem, IV oxacillin, PO ceftibuten and IV ceftriaxone + metronidazole. Three patients presented severe neutropenia, with an ANC <500/mmc. Figure 1 shows the median of the pre-and post-therapy neutrophils (%, n/mmc) and WBC levels (n/mmc). Neither leukopenia, thrombocytopenia nor anemia were observed in the treated patients. None of the patients presented symptoms associated to neutropenia, nor clinical reasons to discontinue antibiotic therapy. Data on resolution of neutropenia are not available, as patients were discharged before full normalization of ANC. Table 1 provides demographic characteristics of the included patients, details on antibiotic therapy, in terms of drug and indication for use and variations into ANC and WBC count. Characteristics of antibiotic courses and neutropenia’s TTO are reported in the Supplementary Table S2.

**TABLE 1:** Anagraphic characteristics of the included patients, details on antibiotic therapy, in terms of drug and indication use, and variations into ANC and white blood cells (WBC) count.

Case	Age (months)	Sex	Diagnosis	Antibiotic (Route of administration)	WBC T0 (n/mmc)	Neutrophils T0 (ANC, %)	Time to onset (days)	WBC TX (n/mmc)	Neutrophils TX (ANC, %)	Symptoms	Therapy discontinued
1	25	F	Osteomyelitis	Oxacillin (IV)	14660	7138 (49%)	27	4370	1000 (23%)	No	No
2	5	M	UTI	Ceftriaxone (IV)	17180	13056(76%)	3	6220	1030 (16%)	No	No
3	22	F	UTI	Amoxicillin/Clavulanic Acid (PO)	7380	3025(41%)	3	4410	350 (8%)	No	No
4	2	M	UTI	Ceftriaxone (IV)	10200	4896(48%)	6	9880	1230 (12%)	No	No
5	12	F	UTI	Ceftibuten (PO)	6670	3068(46%)	4	13740	960 (7%)	No	No
6	2	F	UTI	Amoxicillin/Clavulanic Acid (PO)	10170	4983(49%)	2	6100	980 (16%)	No	No
7	3	M	UTI	Ceftriaxone (IV)	29740	18141(61%)	3	5040	520 (10%)	No	No
8	3	M	UTI	Amoxicillin/Clavulanic Acid	16530	6612(40%)	4	15740	1430 (9%)	No	No
9	3	M	Soft tissues infection	Ceftriaxone (IV)	15170	6068(40%)	4	9400	410 (4%)	No	No
10	6	F	UTI	Amoxicillin/Clavulanic Acid (PO)	7380	4649(63%)	5	7960	420 (5%)	No	No
11	2	F	UTI	Ceftriaxone (IV)	13790	6619(48%)	3	10480	1150 (11%)	No	No
12	8	F	UTI	Ceftriaxone (IV)	25800	14706(57%)	5	8110	1370 (17%)	No	No
13	51	M	Osteomyelitis	Ampicillin/Sulbactam (IV)	10260	6258(61%)	14	3950	1190 (30%)	No	No
14	24	F	UTI	Meropenem (IV)	9820	4050(41%)	5	8950	1280 (14%)	No	No
15	18	F	Soft tissues infection	Ceftriaxone (IV) + Metronidazole (IV)	13390	4050(48%)	6	8990	1490 (16%)	No	No
16	1	M	UTI	Ampicillin Sulbactam (IV)	9880	5640(58%)	2	6330	730 (11%)	No	No

ANC, Absolute Neutrophil Count; F,Female; IV, Intravenous; M,Male; PO, Per Os (oral); UTI, Urinary Tract Infection; T0, Time of diagnosis; TX, Detection time of neutropenia; WBC, White Blood Cells.

### 3.3 Systematic Review

**FIGURE 2:**
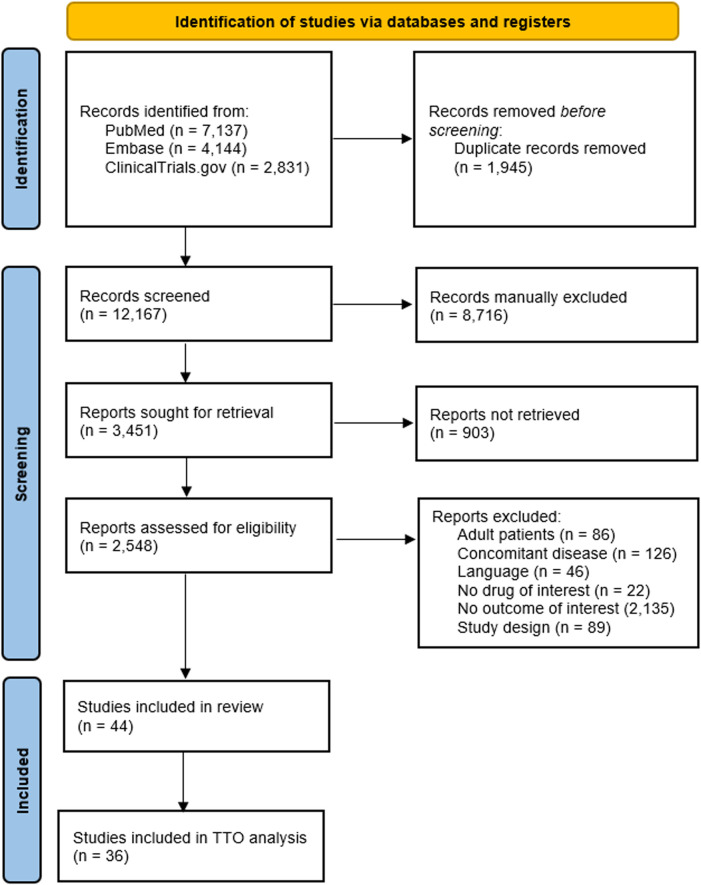
The PRISMA flowchart.

The study selection and screening process is presented in the PRISMA flowchart (Figure 2). Out of the 12,167 unique titles retrieved (7,137 articles from PubMed, 4,144 from Embase, and 2,831 from Clinical.Trials.gov), 2,548 full-text articles were found and assessed for eligibility. Ultimately, 44 studies fulfilled our inclusion criteria. Supplementary Table S3 summarizes the characteristics of all the studies included in the systematic review (including the related references).

The retrieved studies comprise case reports or case series (*n* = 13), prospective observational studies (*n* = 11), randomized controlled studies (*n* = 10), retrospective observational studies (*n* = 5), and non-randomized clinical trials (*n* = 5), published between 1962 and 2020, and reporting on a total of 2,602 pediatric patients treated with antibiotics.

Administered antibiotics were mainly penicillins alone (*n* = 35 studies; amoxicillin, ampicillin, cloxacillin, flucloxacillin, methicillin, nafcillin, oxacillin, piperacillin) or associated with β-lactamase inhibitors (*n* = 7; amoxicillin/clavulanic acid, piperacillin/tazobactam, ticarcillin/clavulanic acid), followed by cephalosporins (*n* = 12; cefaclor, cefepime, cefetamet pivoxil, cefixime, cefoxitin, ceftriaxone), macrolides (*n* = 7; azithromycin, clarithromycin, roxithromycin), sulphonamides (*n* = 7; sulfadimethoxine, trimethoprim/sulfamethoxazole), glycopeptides (*n* = 2; teicoplanin, vancomycin), and others (chloramphenicol, *n* = 2; moxalactam, *n* = 2; dapsone, *n* = 1; daptomycin, *n* = 1; imipenem/cilastatin, *n* = 1; linezolid, *n* = 1; rifabutin, *n* = 1).

These antibiotics were mostly used to treat otitis (*n* = 16), osteomyelitis (*n* = 9), pneumonia (*n* = 8), cellulitis (*n* = 7), bone/joint infections (*n* = 7), and bacterial meningitis (*n* = 7).

In all studies, treatment lasted less than 2 months, apart from an observational study (Losurdo et al., 1998) that reported 6 months of rifabutin and clarithromycin administration, and a clinical trial that investigated trimethoprim/sulfamethoxazole use for UTI prophylaxis, for which the drug administration ranged from 6 to 50 months (Hori et al., 1997).

A total of 228 pediatric patients experienced neutropenia following antibiotic administration: 77 patients required antibiotic withdrawal following neutropenia occurrence (and one dose reduction), while in all others cases it resolved spontaneously. All cases resolved within 2 months, apart from one patient reported by Al-Fadley et al., who was treated with ampicillin/cloxacillin for 26 days and required 95 days after withdrawal to reach a neutrophils level of 1,500 cells/mmc; and one patient treated with amoxicillin for 10 days took 86 days for his neutropenia to resolve (Feldman et al., 1985).

Neutropenia was rarely accompanied by complications, such as rash and fever. Associated adverse events observed in patients experiencing neutropenia were mainly eosinophilia, thrombocytopenia and elevation of liver enzymes.

TTO was measured for 215 patients (extracted from 36 studies); details concerning studies included in the TTO analysis are reported in Table 2.

**TABLE 2:** Details of neutropenia occurrence in studies included in TTO analysis.

Study	Main author (year)	Antibiotic [ATC Code]	n patients with neutropenia (% out of total patients)	n neutrophils/mmc	TTO (days)	Antibiotic withdrawal	Resolution (days)	Symptoms or complications	Other associated ADR
1	Jarkowski TL and Martmer EE (1962)	Sulfadimethoxine [J01ED01]	1 (100)	914	5	Y	NA	Toxic epidermal necrosis	Death
2	Leventhal JM and Silken AB (1976)	Oxacillin [J01CF04]	1 (100)	0	19	Y	2	NA	NA
		Oxacillin [J01CF04]	1 (100)	297	19	Y	4	N	NA
		Oxacillin [J01CF04]	1 (100)	560	19	Y	4	N	NA
3	Chu JY, et al. (1977)	Oxacillin, ampicillin [J01CF04, J01CA01]	1 (100)	0	17	Y	5	N	N
4	Greene GR and Cohen E (1978)	Nafcillin [J01CF06]	1 (100)	600	24	Y	6	N	NA
		Nafcillin [J01CF06]	1 (100)	690	4	Y	3	N	NA
5	Ardati KO, et al. (1979)	Trimethoprim, sulfamethoxazole [J01EE01]	9 (50)	480 (1 pt); 560 (1 pt); 1,150-1,420 (7 pt)	4 (3 pt); 7 (1 pt); 11 (1 pt); 12 (1 pt); 23 (1 pt); NA (2 pt)	Y (2 pt)	NA	N	Eosinophilia, thrombocytopenia, transient elevation of liver enzymes
6	Feldman WE, et al. (1980)	Cefoxitin [J01DC01]	2 (11)	<1,000	9 (1 pt); R: 3-21 (1 pt)	Y (1 pt)	2 (1 pt)	N	Eosinophilia
7	Asmar BI, et al. (1981)	Trimethoprim/ Sulfamethoxazole [J01EE01]	17 (34)	344 (1 pt); <750 (7 pt); <1,200 (7 pt)	M: 5.8	Y (1 pt)	M: 8.9; R: 3-23	N	Eosinophilia, thrombocytopenia, anemia
		Amoxicillin J01CA04	1 (5)	1,309	10	N	NA	N	Eosinophilia
8	Dutro MP, et al. (1981)	Nafcillin [J01CF06]	1 (100)	54	22	Y	4	N	N
		Nafcillin [J01CF06]	1 (100)	252	9	Y	3	N	N
9	Kumar K and Kumar A (1981)	Ampicillin [J01CA01]	1 (100)	156	10	N	16	Fever	NA
		Chloramphenicol, ampicillin [J01BA01, J01CA01]	1 (100)	0	14	Y	25	NA	NA
10	St John MA and Prober CG (1981)	Cloxacillin [J01CF02]	2 (3)	<500	2 (1 pt); 10 (1 pt)	N	NA	N	Eosinophilia, elevated liver enzymes
11	Tuomanen EI, et al. (1981)	Chloramphenicol [J01BA01]	11 (25)	<1,000	M: 8.2, R: 7-10 (5 pt); 45 (4 pt); 120 (1 pt); 330 (1 pt)	Y	1-2 (5 pt); 7 (4 pt); 14 (1 pt); 21 (1 pt)	N	N
12	Kaplan SL, et al. (1983)	Moxalactam [J01DD06]	20 (53)	<500 (1 pt); <1,000 (10 pt); <1,500 (8 pt)	R: 1-21; 5 (11 pt); 21 (9 pt)	N	NA	N	Eosinophilia, thrombocytopenia
13	Chonmaitree T, et al. (1984)	Ceftriaxone [J01DD04]	2 (4)	390 (1 pt); 616 (1 pt)	R: 5-14; 5 (1 pt); 14 (1 pt)	Y	R: 2-7	N	Thrombocytosis, eosinophilia
14	Dubs MMA (1985)	Amoxicillin/clavulanic acid, ticarcillin/clavulanic acid [J01CR02, J01CR03]	1 (100)	212	23	Y	5	N	N
15	Feldman S, et al. (1985)	Trimethoprim/sulfamethoxazole [J01EE01]	28 (57)	<1,500	M: 18.4 ± 4.3; R: 10-23	NA	R: 23-37	N	Thrombocytopenia, hemoglobin decrease
		Amoxicillin [J01CA04]	22 (54)	<1,500	M: 17.8 ± 4.4	NA	R: 23-86	N	Thrombocytopenia, hemoglobin decrease
16	Higham M, et al. (1985)	Ceftriaxone [J01DD04]	2 (6)	150 (1 pt); 700 (1 pt)	6 (1 pt); 3 (1 pt)	Y	7 (1 pt); NA (1 pt)	N	NA
17	Schaad UB, et al. (1987)	Amoxycillin/clavulanic acid [J01CR02]	1 (1)	248	6	N	21	N	Elevated liver enzymes
18	Ahonkhai VI, et al. (1989)	Imipenem/cilastatin [J01DH51]	4 (2)	<1,000	M: 6.3; N: 5; R: 1-26	N	NA	N	NA
19	Al-Fadley F (1992)	Ampicillin, cloxacillin [J01CA01, J01CF02]	1 (100)	950	15	Y	8	Rash, fever	N
		Ampicillin, cloxacillin [J01CA01, J01CF02]	1 (100)	340	15	Y	95	Rash, fever	N
		Ampicillin, cloxacillin [J01CA01, J01CF02]	1 (100)	620	14	Y	38	Rash	Eosinophilia
		Cloxacillin [J01CF02]	1 (100)	230	23	Y	25	Rash, fever	Eosinophilia
		Cloxacillin, piperacillin [J01CF02, J01CA12]	1 (100)	1,400	20	Y	41	Rash, fever	Eosinophilia
20	Dagan R, et al. (1994)	Ceftriaxone, cefetamet pivoxil [J01DD04, J01DA26]	1 (2)	NA	7	N	NA	N	N
21	Shinohara YT and Colbert J (1994)	Vancomycin [J01XA01]	1 (100)	990	15	Y	NA	N	NA
22	Bégué P and Astruc J (1995)	Roxithromycin [J01FA06]	5 (1)	500-1,000	M: 9 (4 pt); 2 (1 pt)	Y (2 pt)	1 (1 pt); 4 (1 pt)	NA	NA
23	Arguedas A, et al. (1996)	Amoxicillin/clavulanic acid, [J01CR02]	3 (7)	<1,500	10	N	NA	N	N
24	Arguedas A, et al. (1997)	Azithromycin [J01FA10]	2 (6)	<1,500	3	N	NA	NA	NA
		Clarithromycin [J01FA09]	3 (6)	<1,500	10	N	NA	NA	NA
25	Hori C, et al. (1997)	Trimethoprim/sulfamethoxazole[J01EE01]	2 (6)	<1,000	365	N	NA	N	N
26	Losurdo G, et al. (1998)	Rifabutin, clarithromycin [J04AB04, J01FA09]	3 (43)	NA	M: 21; R: 14-28	N (dose reduction)	5	N	NA
27	Kaplan SL, et al. (2001)	Linezolid [J01XX08]	5 (8)	58 (1 pt); 1,020 (1 pt); 1,150 (1 pt); 1,370 (1 pt); 1,470 (1 pt)	3 (3 pt); M: 12.2, R: 6-41 (2 pt)	Y	11	N	NA
28	Wee IY and Oh HM (2001)	Teicoplanin [J01XA02]	1 (100)	706	14	Y	7	Rash, fever	Elevated liver enzymes, rash
29	Arguedas A, et al. (2003)	Azithromycin [J01FA10]	19 (14)	<1,500	14	N	NA	NA	NA
		Ceftriaxone [J01DD04]	8 (13)	<1,500	14	N	NA	NA	NA
30	Jacobs RF, et al. (2005)	Azithromycin [J01FA10]	2 (6)	1,400 (1 pt); 1,500 (1 pt)	3	N	36	N	N
31	Pietroni M (2005)	Cloxacillin [J01CF02]	1 (100)	140	34	Y	NA	Fever	Sepsis, death
32	Van Den Boom J, et al. (2005)	Flucloxacillin [J01CF05]	8 (100)	M: 710; R: 190-1,250	15 (1pt); 20 (3 pt); 25 (1 pt); 27 (1 pt); 36 (1 pt); 58 (1 pt)	Y	5.6	Rash, fever	NA
33	Hettmer S and Heeney MM (2008)	Cefepime [J01DE01]	1 (100)	20	19	Y	5	NA	NA
34	Yusef D, et al. (2017)	Piperacillin/Tazobactam [J01CR05]	10 (26)	<1,500	Median: 18	Y	NA	NA	Fever, elevated liver enzymes, elevated CRP, abdominal pain
35	Patel S, et al. (2018)	Ceftriaxone [J01DD04]	1 (1)	NA	21	Y	NA	Rash, fever	NA
36	Fernando M, et al. (2019)	Dapsone [J04BA02]	1 (100)	0	60	Y	5	Skin sepsis	Leukopenia, bite cells, blister cells, agranulocytosis

ADR, Adverse Drug Reaction; ATC, Anatomical Therapeutic Chemical; M, mean; N, no; n, number; NA, not available; pt, patients; R, range; TTO, Time To Onset; Y, yes. The detailed list of references is reported in Supplementary Table S3

Fifty-seven patients (8 studies) were excluded from the TTO analysis because a precise date of neutropenia occurrence was not provided. Details of neutropenia occurrence reported in the studies excluded from TTO analysis are reported in Supplementary Table S4.

### 3.4 Time-to-Onset Analysis

We analysed (a total of) 281 antibiotic-related neutropenia events and 11 categories of antibiotics (Table 3). In general, the retrospective chart review (*n* = 16) detected neutropenia during the first days of treatment [median days (25th–75th percentiles) = 4 (3–5)], while data from the literature (*n* = 224) and FAERS (*n* = 41) show longer times [median days (25th–75th percentiles), 15 (9–18) vs. 10 (6–18) for literature and FAERS, respectively]. Median TTO ranged from 4 to 60 days after the start of the therapy. For ATC class J01X, J01F, J01E and J04A median TTOs retrieved from more than one source revealed high accordance (*p* > 0.05) with J01X causing neutropenia in less than a week and J01F/J01E/J04A in more than 10 days. On reverse, J01D and J01C were discordant among resources (*p* < 0.05), with a reduced median TTO (less than 7 days) in patients included in the retrospective chart review.

**TABLE 3:** Comparisons of TTO analyses among findings from pharmacovigilance study, clinical observation and literature.

ATC Class (WHO)	Retrospective chart review		Systematic review		FAERS		*p* ^a^
	*n*	TTO (days)	n	TTO (days)	*n*	TTO (days)	
J01X: Other antibacterials	-	-	7	12 (3–13)	4	4 (3–8)	>0.05
J01G: Aminoglycoside antibacterials	-	-	-	-	3	7 (6–92)	-
J01D: Other beta-lactam antibacterials	9	4 (3–5)	42	11 (5–18)	7	9 (6-15)	<0.05
J01M: Quinolone antibacterials	-	-	-	-	5	9 (8–14)	-
J01F: Macrolides, lincosamides and streptogramins	-	-	34	14 (9–14)	3	10 (10 -10)	>0.05
J01E: Sulfonamides and trimethoprim	-	-	55	18 (6–18)	3	10 (10–20)	>0.05
J01C: Beta-lactam antibacterials, penicillins	7	4 (3-10)	70	18 (16–19)	8	14 (6–16)	<0.05
J04A: Drugs for treatment of tuberculosis	-	-	3	21 (21-21)	7	16 (9–32)	>0.05
J02A: Antimycotics for systemic use	-	-	-	-	1	22	-
J01B: Amphenicols	-	-	12	30 (10–45)	-	-	-
J04B: Drugs for treatment of lepra	-	-	1	60	-	-	-
Total	16	4 (3–5)	224	15 (9–18)	41	10 (6–18)	

n, number of cases of neutropenia related with an antibiotic of that class; TTO, time to onset, median (25th-75th percentiles).

a Wilcoxon-Mann-Whtiney for comparison between two groups and ANOVA Kruskall-Wallis for three groups.

Unfortunately, some ATC classes were only available from one resource: a median TTO of 7 days was reported for J01G (*n* = 3), 9 days for J01M (*n* = 5), 22 days for J02A (*n* = 1), 30 days for J01B (*n* = 12), 60 days for J04B (*n* = 1).

## 4 Discussion

Antibiotic-associated neutropenia is a relatively uncommon AE with a largely variable prevalence in the pediatric age (Gomez et al., 2001; Maraqa et al., 2002; Olson et al., 2015). Clinicians’ awareness about common and uncommon antibiotic-related AEs, including neutropenia, is of great importance to improve quality of healthcare for children. The integrated approach based on pharmacoepidemiological analyses and a systematic review of all the currently available pediatric evidence used for this review allow us to provide detailed characterization in terms of distribution of agent groups, duration and treatment options, seriousness, among which the temporal association between the wide spectrum of antibiotic options and this rare but potentially clinically relevant adverse event. The first important finding is on TTO as data available on this matter in pediatric population are still scarce, especially regarding shorter and oral antibiotic therapy. In fact, most of the evidence available in pediatric age derives from studies on complications of outpatient, long-term, parenteral antibiotic therapy, as the those conducted by Olson et al. and Gomez et al., which found, respectively, a TTO of 21 and 20 days since the beginning of therapy (Gomez et al., 2001; Olson et al., 2015).

Our analysis with an integrated approach, reveals that the median TTO of antibiotic-induced neutropenia in the pediatric population is in fact shorter than previously reported (Gomez et al., 2001; Olson et al., 2015; Holz et al., 2021). The most discordant datum was found in the retrospective chart review, where median TTO we found to be 4 days. Different aspects should be taken into account for explaining this observation. All patients included in the case series analysis were infants or young children (median age 5 months), and all were hospitalized for acute and relatively severe infections. In these patients, a stricter follow-up through repeated assessments of laboratory values has allowed an earlier detection of neutropenia. Also in the pharmacovigilance study, median TTO was 10 days, that was, again, a much shorter period respect to findings of previous reports. As FAERS data extraction was limited to ICSRs reported by physicians, an easier access to laboratory assessments and a generally greater attention to ADRs may also in this case explain this datum. We cannot exclude that the reports of AEs may have been carried out by clinicians with greater awareness of potential ADRs and are more prone to strictly monitoring them. Indeed, in the systematic review analysis, neutropenia median TTO was 15 days, in line with the only previous study (Gomez et al., 2001; Olson et al., 2015; Holz et al., 2021); this may be also due to the type of included studies (mainly retrospective and case series), hence the heterogeneity of patients (different age range), settings (inpatient and outpatient) and severity of treated infections. As for the antimicrobial agent groups, the shortest TTOs were seen in patients treated with ß-lactam antibacterials, other than penicillins (ATC code J01D) in all the analyses we carried out (case series, pharmacovigilance study and systematic review).

However, we found a significantly shorter TTO in the chart review respect to FARES reports and systematic review (4 vs. 9 vs. 11 days). On the contrary, both in systematic review and FAERS analysis, longer TTO were detected in patients treated with antituberculars (21 and 16 days, respectively).

As for the distribution of antimicrobial agent groups, we found that, penicillins and other ß-lactams, followed by sulphonamides and macrolides, resulted to be more frequently associated to neutropenia; similarly, in the case series, patients who developed neutropenia were mostly treated with cephalosporins and ß-lactams. Penicillins and other ß-lactams are the most widely prescribed antibiotics in pediatric age, either orally or intravenously. Consequently, AEs in general, and neutropenia specifically, may be more frequently detected with the use of these antimicrobial drug classes. This finding is in contrast to what Olson et al. (2015) observed in their systematic review, in which they did not find an association between neutropenia and ß-lactams antimicrobials, even if the majority of detected AEs occurred in patients treated with cephalosporines. The 41 neutropenia cases reported to FAERS resulted to be much more distributed among the different antimicrobial classes; once again, this observation may be result of a greater attention by the clinicians who are more familiar with AEs reporting. Recent evidence in adult population (Holz et al., 2021) found neutropenia to be mostly observed in patients treated with vancomycin and ceftaroline; however, these drugs are not so frequently prescribed in children.

It is well-known that drug-drug interactions (DDIs) may exacerbate ADRs, including antibiotic-associated neutropenia. In the retrospective chart review, we excluded patients treated with drugs other than antibiotics and all children, apart one, received an antibiotic monotherapy. On the contrary, in 88.46% of cases, patients included in FAERS analysis received more than one antibiotic as well as other concomitant treatments; only in two cases pharmacokinetic DDIs might have increased the risk for neutropenia (Supplementary Table S2) (Drug Interaction, 2022).

The consequences of antibiotic-associated neutropenia may vary in severity (Bhatt and Saleem, 2004; Segel and Halterman, 2008; Holz et al., 2021); in the pediatric age most cases are asymptomatic and resolve spontaneously without any treatment (Gomez et al., 2001; Barg et al., 2015; Celkan and Koç, 2015). In our case series, all neutropenia cases were asymptomatic and none of them required leukopoiesis stimulants, withdrawal of therapy, dose reduction or prolongation of hospitalization, differently to what we have observed in the systematic review, where antibiotics were discontinued in nearly 34% of cases (without requiring any treatment).

In FAERS reports, half of the patients experiencing neutropenia were hospitalized. However, only three presented neutropenia alone; in all other cases, systemic symptoms and other laboratory abnormalities were also present and may have contributed to the need of hospitalization. Moreover, FAERS reports likely include more severe ADRs. Although benign in the majority of cases (Segel and Halterman, 2008), antibiotic associated neutropenia should not be neglected as, even if rarely, the AE may put children at higher risk of severe infections and, in few serious cases, may also be fatal. Finally, development of antibiotic-associated neutropenia may result in discontinuation or modification of the ongoing and causative treatment with a potentially negative impact on the overall efficacy of therapy.

### 4.1 Strengths and Limitations

This is the first study aimed at specifically investigating the occurrence of neutropenia following antibiotic treatment that may be used to improve of antibiotic-induced neutropenia recognition in the pediatric clinical setting.

The use of a spontaneous reporting system database has some important implicit limitations because reporting is influenced by factors such as notoriety bias [media attention and recent publication of an adverse drug reaction in the literature might stimulate the reporting trend activity (Teng and Frei, 2022)], selection bias and under-reporting (Faillie, 2019).

Moreover, the quality of information, including the grade of completeness, may be suboptimal leading to misclassification bias. The certain causal correlation and incidence rates cannot be determined from the FAERS since the primary goal of spontaneous reporting systems is to signal the existence of a possible relationship between a drug or drug class and an adverse event, without proving any causality or providing the denominator (drug exposure). With regard to the case series, it is worth mentioning that the period of observation (one and a half year) is not long enough to detect the events of low incidence. The attempts to minimize these potential biases, we included high-quality reports (providing all relevant information such as sex, age, drug name, indication use, concomitant therapy, start and event date) sent by physicians only. Furthermore, when selecting the patient cohort from the three sources, we took into account the same inclusion/exclusion criteria to avoid confounding factors regarding neutropenia’s etiology.

## 5 Conclusion

Our analysis extends previous evidence on the occurrence of neutropenia in the pediatric clinical setting providing precise information on the temporal association between the wide spectrum of antibiotic options and this uncommon adverse event, thus supporting its early identification and eventual management.

The integrated approach based on pharmacoepidemiological analyses and a systematic review of all the currently available pediatric evidence suggest that a stricter follow-up through repeated assessments of laboratory values is of crucial importance for an earlier detection of neutropenia (median TTO: 4 days). The shortest TTOs were seen in patients treated with other than penicillins ß-lactams in either the case series, pharmacovigilance study and systematic review (range: 4–11 days); in contrast, longer TTO were detected in patients treated with antituberculars (16 > days).

More importantly, antibiotics were discontinued in nearly 34% of pediatric cases detected in the literature; in FAERS reports, half of the patients experiencing neutropenia (along with systemic symptoms and other laboratory abnormalities) were then hospitalized. Given the potential clinical consequences of this rare but potentially life-threatening event, continuous attention for this side effect with an appropriate monitoring are warranted in pediatric patients receiving antibiotic-based therapy.

## Data Availability

The original contributions presented in the study are included in the article/Supplementary Material, further inquiries can be directed to the corresponding author.
