# Supplementary material for: Antibiotic-Induced Neutropenia in Pediatric Patients: New Insights From Pharmacoepidemiological Analyses and a Systematic Review
Source: Front Pharmacol. 2022 Jun 2;13:877932. doi: 10.3389/fphar.2022.877932 (PMC9201445; doi:10.3389/fphar.2022.877932)
Supplement: Supplementary file 1 [file Table1.docx]

**Supplementary Table 1.** Details of all the retrieved cases related to the occurrence of Antibiotic-associated neutropenia, from to the FAERS.

| **Case** | **Age**  **(yrs)** | **Sex** | **Adverse Drug reaction** | **Disease** | **Suspect drugs**  **(subministration Route)** | **Concomitant drugs** | **DDI*** | **Hospitalisations** |
| --- | --- | --- | --- | --- | --- | --- | --- | --- |
| **1** | <1 | F | *Neutropenia***,** rash maculo-papular | Infection | Cefotaxime (IV)  Metamizole (IV) | Acetaminophen | N | Y |
| **2** | <1 | F | *Neutropenia* | Staphylococcal infection,  epilepsy | Clonazepam (oral)  Enoxaparin (subcutaneous)  Flucloxacillin (IV)  Valproic acid (oral)  Gentamicin (IV)  Domperidone (NA)  Amoxicillin\clavulanic acid (IV) | NA | N | Y |
| **3** | <1 | F | Leukopenia, liver function test increased, *neutropenia,* pyrexia,  rash generalised, transaminases increased | Arthritis bacterial, osteomyelitis,  pyrexia | Ceftriaxone (IV) | Ibuprofen,  Acetaminophen | N | Y |
| **4** | 1 | F | Anaemia, *neutropenia,* platelet count decreased | Device related infection | Vancomycin (IV)  Cloxacillin (IV)  Gentamicin (IV) | NA | N | N |
| **5** | 1.5 | M | Drug reaction with eosinophilia and systemic symptoms, enterococcal infection, eosinophilia, inflammatory marker increased**,** *neutropenia,* pyrexia, rash maculo-papular | Meningitis streptococcal, seizure | Cefotaxime (IV)  Phenytoin (IV) | Amoxicillin,  Chloral Hydrate,  Chlorpheniramine,  Fentanyl,  Heparin Sodium,  Levetiracetam,  Rifampin,  Sodium Feredetate,  Teicoplanin | N | Y |
| **6** | 2 | F | Drug rash with eosinophilia and systemic symptoms, eosinophilia, pyrexia, rash maculo-papular, cytolytic hepatitis, aplasia pure red cell, bicytopenia, histiocytosis haematophagic, dermatitis exfoliative**,** *neutropenia* | Infection | Ciprofloxacin (oral)  Piperacillin (IV) | NA | N | Y |
| **7** | 4 | F | *Neutropenia,* viral infection | Bronchitis | Clarithromycin (oral)  Tosufloxacin (oral) | NA | N | N |
| **8** | 8 | M | fatigue, *neutropenia,* pallor | Prophylaxis | Amoxicillin (oral) | Macrogol,  Mometasone | N | N |
| **9** | 8 | F | arthralgia, hypersensitivity, *neutropenia,* pyrexia | NA | Rifampin (oral) | Isoniazid | N | N |
| **10** | 8 | M | *neutropenia,* off label use, product use issue, transaminases increased | Immunisation | Linezolid (oral) | Azithromycin,  Pneumococcal Vaccine Polyvalent 23 | N | N |
| **11** | 9 | F | erythema multiforme, liver function test abnormal, *neutropenia* | Mycobacterium abscessus infection | Linezolid (IV)  Cefoxitin (IV) | Omeprazole,  Macrogol,  Insulin,  Flucloxacillin,  Vitamin E,  Thyroxine,  Ivacaftor,  Multivitamins,  Amylase,  Lipase,  Protease | N | N |
| **12** | 10 | F | lymphopenia, *neutropenia* | Infection | Rifampin (oral)  Sulfamethoxazole\Trimethoprim, (IV)  Fluconazole (IV) | NA | N | N |
| **13** | 10 | M | alanine aminotransferase increased, anaemia, convulsion, drug reaction with eosinophilia and systemic symptoms, general physical health deterioration, hepatocellular injury, lipase increased, *neutropenia,* platelet count decreased, pyrexia, vomiting | Convulsion | Metronidazole (IV)  Levetiracetam (oral) | Cefotaxime,  Lactulose,  Acetaminophen | N | Y |
| **14** | 11 | M | *neutropenia* | Cystic fibrosis, haemoptysis,  anaemia | Amikacin Sulfate (IV)  Ciprofloxacin (IV) | Omeprazole,  Vitamin K1,  Ursodeoxycholic Acid,  Iron,  Acetylcysteine,  Amylase,  Lipase,  Protease,  Dornase Alfa,  Budesonide,  Albuterol | N | Y |
| **15** | 11 | F | leukopenia, pyrexia, asthenia, *neutropenia,* drug interaction, thrombocytopenia, erythema | Mycobacterial infection | Clarithromycin (oral) | Meropenem,  Amikacin,  Ciprofloxacin | N | Y |
| **16** | 11 | F | *neutropenia* | Anticoagulant therapy,  brain empyema | Enoxaparin (subcutaneous)  Metronidazole (IV)  Amoxicillin (IV) | NA | N | N |
| **17** | 12 | M | agranulocytosis, leukopenia, *neutropenia,* pyrexia | Osteomyelitis acute,  pain | Amoxicillin\Clavulanic Acid (IV)  Acetaminophen (oral)  Ibuprofen (oral) | Gentamycin,  Ceftriaxone,  Amikacin | Y  Ibuprofen may increase amikacin exposure by decreasing renal clearance | N |
| **18** | 13 | M | hyperuricaemia, lymphopenia, *neutropenia* | Pulmonary tuberculosis | Rifampin (oral)  Pyrazinamide (oral)  Ethambutol Hydrochloride (oral)  Isoniazid (oral) | NA | N | N |
| **19** | 16 | M | liver function test abnormal, *neutropenia,* rash, thrombocytopenia | Meningitis | Ceftriaxone (IV) | NA | - | Y |
| **20** | 16 | F | cell death, dermatitis allergic, leukopenia, *neutropenia,* thrombocytopenia | Lyme disease | Ceftriaxone (NA) | Dexchlorpheniramine,  Ramipril,  Hydrochlorothiazide,  Paroxetine,  Cefotaxime, Cephradine,  Cephalexin,  Amoxicillin,  Cefuroxime | N | Y |
| **21** | 16 | F | feeling cold, leukopenia, *neutrophil count decreased,* pancytopenia, pyrexia, thrombocytopenia, tremor | Helicobacter gastritis | Rifabutin (NA)  Levofloxacin (NA)  Amoxicillin (NA)  Esomeprazole (NA) | NA | N | Y |
| **22** | 16 | M | leukopenia, *neutropenia,* rash macular | Infection | Levofloxacin (oral)  Sulfamethoxazole\Trimethoprim (oral) | NA | N | N |
| **23** | 16 | M | *neutropenia* | Latent tuberculosis | Isoniazid\Rifampin (oral) | NA | N | N |
| **24** | 16 | F | *neutropenia,* photosensitivity reaction | Pyelonephritis | Cefotaxime (IV) | NA | - | Y |
| **25** | 16 | M | *neutropenia,* purpura, pyrexia, rash maculo-papular, thrombocytopenia | Skin infection | Clarithromycin (oral)  Rifabutin (oral) | NA | Y  Clarithromycin may increase rifabutin exposure via CYP3A4 inhibition | Y |
| **26** | 17 | M | dyspnoea, hypersensitivity, lip swelling, lymph node pain, musculoskeletal stiffness, nausea, *neutropenia,* paraesthesia oral, rash erythematous | Acne | Trimethoprim (oral) | Adapalene,  Lymecycline | N | N |

IV: intravenous; NA: not available; Y: Yes; yrs, years N: no

CYP3A4: cytochrome P450 3A4.

*DDI: Drug-Drug Interaction; DDIs were evaluated by using the UptoDate tool [Lexicomp Drug Interaction. Waltham, MA: UpToDate, Inc.; 2022. https://www.uptodate.com/drug-interactions/?source=responsive_home#di-druglist. Accessed March 29, 2022]
