# Supplementary material for: Antibiotic-Induced Neutropenia in Pediatric Patients: New Insights From Pharmacoepidemiological Analyses and a Systematic Review
Source: Front Pharmacol. 2022 Jun 2;13:877932. doi: 10.3389/fphar.2022.877932 (PMC9201445; doi:10.3389/fphar.2022.877932)
Supplement: Supplementary file 2 [file Table2.docx]

**Supplementary Table 2.** Characteristics of antibiotic courses and neutropenia’s time to onset.

| **Antibiotic**  **(ATC Code)** | **Number of patients treated** | **Infection** | **Route of administration** | **Dose (mg/kg/die)** | **Duration of treatment (time range in days, median in days)** | **Time to onset**  **(time range in days, median in days)** |
| --- | --- | --- | --- | --- | --- | --- |
| Ceftriaxone  (J01DD04) | 6 (37.5%) | UTI,  Soft tissues infection | IV | 50-75 | 5-7 (6) | 3-6 (4) |
| Amoxicillin/Clavulanic Acid  (J01CR02) | 4 (25%) | UTI | PO | 80 | 6-10 (10) | 2-4 (3.5) |
| Ampicillin/Sulbactam  (J01CR01) | 2 (12.4%) | UTI, Osteomyelitis | IV | 200 | 10-21 (15.5) | 2-14 (8) |
| Ceftriaxone(J01DD04) + metronidazole (J01XD01) | 1 (6.2%) | Soft tissues infection | IV | 75 + 22 | 6 | 6 |
| Meropenem  (J01DH02) | 1 (6.2%) | UTI | IV | 60 | 10 | 5 |
| Oxacillin  (J01CF04) | 1 (6.2%) | Osteomyelitis | IV | 200 | 28 | 27 |
| Ceftibuten  (J01DD14) | 1 (6.2%) | UTI | PO | 9 | 10 | 4 |

IV = intravenous. PO = orally
