# Supplementary material for: Antibiotic-Induced Neutropenia in Pediatric Patients: New Insights From Pharmacoepidemiological Analyses and a Systematic Review
Source: Front Pharmacol. 2022 Jun 2;13:877932. doi: 10.3389/fphar.2022.877932 (PMC9201445; doi:10.3389/fphar.2022.877932)
Supplement: Supplementary file 3 [file Table3.docx]

**Supplementary Table 3.** Characteristics of all the studies included in the systematic review.

| **Main author (year)** | **Type of study** | **N patients** | **Age** | **Females (%)** | **Antibiotic** | **Antibiotic indication** | **Antibiotic dose** | **Antibiotic’s route of administration** | **Duration of treatment (days)** |
| --- | --- | --- | --- | --- | --- | --- | --- | --- | --- |
| Jarkowski TL and Martmer EE (1962) | Case report | 1 | 12 yr | 1 (100) | Sulfadimethoxine | Pneumonia | 500 mg/qd | IV | 7 |
| Leventhal JM and Silken AB (1976) | Case series | 1 | 13 yr | 0 | Oxacillin | Septic arthritis | 200 mg/kg/qd | IV | 19 |
|  |  | 1 | 15.5 yr | 0 | Oxacillin | Surgical wound infection | 200 mg/kg/qd | IV | 19 |
|  |  | 1 | 9 mo | 0 | Oxacillin | Septic arthritis | 200 mg/kg/qd | IV | 19 |
| Chu JY, et al. (1977) | Case report | 1 | 1 yr | 0 | Oxacillin, ampicillin | Osteomyelitis and pyogenic arthritis | 150 mg/kg/qd [oxacillin]; 200 mg/kg/qd ampicillin | IV | 17 |
| Greene GR and Cohen E (1978) | Case series | 1 | 8 mo | 0 | Nafcillin | Cellulitis | R: 100-200 mg/kg/qd | IV | 24 |
|  |  | 1 | 3 mo | 0 | Nafcillin | Wound infection | 150 mg/kg/qd | IV | 4 |
| Ardati KO, et al. (1979) | Prospective observational | 18 | M: 4.3 yr;  R: 3 wk-13 yr | 8 (44) | Trimethoprim/  sulfamethoxazole | Lymphadenitis, cellulitis, osteomyelitis, septic arthritis, shunt infection, meningitis | 10/50 mg/kg/qd | IV, PO | M: 10 [IV], 9 [PO];  R: 4-32 [IV], 2-18 [PO] |
| Feldman WE, et al. (1980) | Clinical trial | 19 | M: 19 mo;  R: 3-146 mo | 7 (37) | Cefoxitin | Cellulitis, arthritis, mastoiditis, lymphadenitis, septicemia, abscess, meningitis | 150 mg/kg/qd | IV | R: 3-21 |
| Asmar BI, et al. (1981) | Prospective observational | 50 | M: 4.2 yr;  R: 0.4-12.6 yr | 24 (48) | Trimethoprim/  sulfamethoxazole | Acute otitis media, uncomplicated UTI | 4 mg/kg/bid | PO | 10 |
|  |  | 20 | M: 2.4 yr  R: 0.3-10 yr | 9 (45) | Amoxicillin | Acute otitis media, uncomplicated UTI | 50 mg/kg/qd | PO | 10 |
| Dutro MP, et al. (1981) | Case series | 1 | 7 yr | 1 (100) | Nafcillin | Osteomyelitis | 150 mg/kg/qd | IV | 22 |
|  |  | 1 | 2 yr | 1 (100) | Nafcillin | Pleural effusion in septicemia | 150 mg/kg/qd | IV | 10 |
| Kumar K and Kumar A (1981) | Case series | 1 | 6 mo | 1 (100) | Ampicillin | Meningitis | 150 mg/kg/qd | IV | 14 |
|  |  | 1 | 15 mo | 0 | Chloramphenicol, ampicillin | Meningitis | 100 mg/kg/qd [chloramphenicol]; 400 mg/kg/qd [ampicillin] | IV | 3 [chloramphenicol];  14 [ampicillin] |
| St John MA and Prober CG (1981) | Retrospective observational | 79 | M: 5 yr;  R: 2 wk-16.5 yr | 26 (33) | Cloxacillin | Skin and soft tissues infections, bone and joints infection, CNS infections | R: 100-200 mg/kg/qd | IV, PO | M: 14;  R: 5-56 |
| Tuomanen EI, et al. (1981) | RCT | 44 | M: 20 mo;  R: 2 mo-7 yr | NA | Chloramphenicol | Bacterial meningitis | 75-100 mg/kg/qd | IV, PO | >9 |
| Kaplan SL, et al. (1983) | Prospective observational | 38 | R: 4-27 mo | NA | Moxalactam | Cellulitis, pneumonia, septic arthritis | R: 113-200 mg/kg/qd | IV, PO | R: 3-21 |
| Chonmaitree T, et al. (1984) | Prospective observational | 48 | M: 3.9 yr;  N: 1 yr;  R: 1 mo-17 yr | 24 (50) | Ceftriaxone | Meningitis, pneumonia, epiglottitis, cellulitis, osteomyelitis, sepsis, septical arthritis, mastoiditis, UTI wound infection, V-P shunt infection | 100 mg/kg/qd [meningitis]; 50-75 mg/kg/qd [other infections] | IV, IM | R: 1-21 |
| Dubs MMA (1985) | Case Report | 1 | 14 mo | 1 (100) | Amoxicillin/  clavulanic acid, ticarcillin/  clavulanic acid | Osteomyelitis | 50/5 mg/kg/qd [amoxicillin/clavulanic acid]; 300/20 mg/kg/qd [ticarcillin/clavulanic acid] | IV | 23 |
| Feldman S, et al. (1985) | Retrospective observational | 49 | R: 2-102 mo | 30 (61) | Trimethoprim/  sulfamethoxazole | Acute otitis media | 8 mg/kg/qd | PO | 10 |
|  |  | 41 | R: 2-102 mo | 25 (61) | Amoxicillin | Acute otitis media | 25 mg/kg/qd | PO | 10 |
| Higham M, et al. (1985) | Prospective observational | 34 | N: 2 yr;  R: 2 mo–14 yr | 10 (29.4) | Ceftriaxone | Abscess, cellulitis, osteomyelitis, pyelonephritis, otitis | 50 mg/kg/qd | IV | R: 3-22 |
| Schaad UB, et al. (1987) | Retrospective observational | 71 | R: 1 mo-16 yr | 26 (37) | Amoxycillin/  clavulanic acid | Peritonsillar abscess, purulent tracheitis, pneumonia, cellulitis, mastoiditis, pyelonephritis | 110 mg/kg/qd [IV]; 50-100 mg/kg/qd [PO] | IV, PO | R: 5-25 |
| Ahonkhai VI, et al. (1989) | Prospective observational | 178 | M: 3.9 yr:  N: 2.9 yr;  R: 26 d – 18 yr | 67 (38) | Imipenem/  cilastatin | Bacterial infection | R: 60-100 mg/kg/qd | IV | R: 1-26 |
| Al-Fadley F (1992) | Case series | 1 | 9 yr | 0 | Ampicillin, cloxacillin | Bone infection | 130 mg/kg/qd [ampicillin]; 130 mg/kg/qd [cloxacillin] | IV | 15 |
|  |  | 1 | 11 yr | 0 | Ampicillin, cloxacillin | Soft tissue infection | 220 mg/kg/qd [ampicillin]; 110 mg/kg/qd [cloxacillin] | IV | 26 |
|  |  | 1 | 2 wk | 0 | Ampicillin, cloxacillin | Soft tissue infection | 145 mg/kg/qd [ampicillin]; 145 mg/kg/qd [cloxacillin IV]; 215 mg/kg/qd [cloxacillin PO] | IV, PO | 28 |
|  |  | 1 | 2 yr | 1 (100) | Cloxacillin | Bone infection | 200 mg/kg/qd | IV | 25 |
|  |  | 1 | 3 yr | 1 (100) | Cloxacillin, piperacillin | Bone infection | 90 mg/qd  [cloxacillin];  135 mg/qd [piperacillin] | IV | 28 |
| Dagan R, et al. (1994) | RCT | 59 | N: 24 mo;  R: 9-60 mo | 21 (36) | Ceftriaxone, cefetamet pivoxil | Pneumonia | 50 mg/kg [ceftriaxone], 20 mg/kg/qd [cefetamet] | IV [ceftriaxone], PO [cefetamet] | 1 [ceftriaxone], 6 [cefetamet] |
| Shinohara YT and Colbert J (1994) | Case report | 1 | 2 yr | 0 | Vancomycin | Endocarditis | 250 mg/qid | IV | 17 |
| Bégué P and Astruc J (1995) | Clinical trial | 477 | M: 4.75 yr  R: 2 mo-15 yr | 202 (42) | Roxithromycin | Lower respiratory tract infections, upper respiratory tract infections, skin and soft tissue infections | 6 mg/kg/qd | PO | M: 9 |
| Arguedas A, et al. (1996) | RCT | 45 | M: 54.4 mo;  R: 4-144 mo | 19 (42) | Amoxicillin/  clavulanic acid | Acute otitis media | 40 mg/kg/qd | PO | 10 |
| Arguedas A, et al. (1997) | RCT | 50 | M: 50 mo;  R: 9-132 mo | 26 (52) | Azithromycin | Acute otitis media | 10 mg/kg/qd | PO | 3 |
|  |  | 47 | M: 50.4 mo;  R: 7-139 mo | 24 (51) | Clarithromycin | Acute otitis media | 15 mg/kg/qd | PO | 10 |
| Hori C, et al. (1997) | Clinical trial | 35 | N: 5 mo;  R: 1-109 mo | 11 (31) | Trimethoprim/  sulfamethoxazole | UTI prophylaxis | 1/5 mg/kg/qod | PO | M: 687 ± 351;  R: 180-1500 |
| Losurdo G, et al. (1998) | Prospective observational | 7 | M: 3.5 yr;  R: 2-8 yr | 6 (85.7) | Rifabutin, clarithromycin | Non-tuberculous mycobacteria lymphadenitis | 5-10 mg/kg/qd [rifabutin];  15 mg/kg/qd [clarithromycin] | PO | 180 |
| Kaplan SL, et al. (2001) | Clinical trial | 66 | N: 3 yr;  R: 1-12 yr | 30 (45.5) | Linezolid | Community acquired pneumonia | 10 mg/kg/bid | IV, PO | M: 12.2 ± 6.2;  R: 6-41 |
| Wee IY and Oh HM (2001) | Case report | 1 | 10 yr | 0 | Teicoplanin | Infective discitis | 300 mg/qd | IV | 14 |
| Arguedas A, et al. (2003) | RCT | 66 | M: 2.5 ± 1.3 yr | 27 (41) | Azithromycin | Acute otitis media | 30 mg/kg | PO | 1 |
|  |  | 66 | M: 2.7 ± 1.5 yr | 30 (45) | Azithromycin | Acute otitis media | 10 mg/kg/qd | PO | 3 |
|  |  | 66 | M: 2.3 ± 1.4 yr | 33 (50) | Ceftriaxone | Acute otitis media | 50 mg/kg | IM | 1 |
| Jacobs RF, et al. (2005) | Prospective observational | 32 | M: 6.7 ± 5.0 yr | 21 (65.6) | Azithromycin | Upper and lower tract respiratory infections | 10 mg/kg | IV | 1 |
| Pietroni M (2005) | Case report | 1 | 11 yr | 1 (100) | Cloxacillin | Chronic osteomyelitis | 500 mg/qid | IV | 42 |
| Van Den Boom J, et al. (2005) | Retrospective observational | 8 | R: 1 mo-13 yr | 2 (25) | Flucloxacillin | Osteomyelitis, septic arthritis | 200 mg/kg/qd | IV | M: 28,1;  R: 19-46 |
| Hettmer S and Heeney MM (2008) | Case report | 1 | 16 yr | 1 (100) | Cefepime | Serratia marcescens wound infection | 113 mg/kg/qd | IV | 19 |
| Yusef D, et al. (2017) | Retrospective observational | 38 | R: 2 mo-17.9 yr | NA | Piperacillin/  tazobactam | Appendicitis, intra-abdominal abscess, bone joint infection, complicated pneumonia | R: 240-300 mg/kg/qd | IV | N: 18;  R: 7-30 |
| Patel S, et al. (2018) | Prospective observational | 123 | R: 0-18 yr | 57 (46.3) | Ceftriaxone | Osteomyelitis, septic arthritis, pulmonary infections, CNS infections, bacterial endocarditis, surgical wound infections | NA | IV | N: 9.2;  IQR: 7.6-19 |
| Fernando M, et al. (2019) | Case report | 1 | 7 yr | 1 (100) | Dapsone | Leprosy | NA | PO | 60 |
| Kitzing W, et al. (1981) | RCT | 25 | M: 6.4 yr | 13 (52) | Methicillin | Bone and joint infection | 200 mg/kg/qd | IV | N: 21;  R: 21-39 |
|  |  | 24 | M: 6.2 yr | 12 (50) | Nafcillin | Bone and joint infection | 150 mg/kg/qd | IV | N: 21  R: 21-22 |
| Keyserling H, et al. (1982) | Prospective observational | 34 | M: 3.3 yr;  R: 2.5 mo-15 yr | 18 (52.9) | Moxalactam | Bacterial infection | 150 mg/kg/qd | IV | M: 7.1;  R: 3-15 |
| Principi N, et al. (1984) | RCT | 40 | M: 2.6 ± 1.7 yr | 18 (45) | Trimethoprim/  sulfamethoxazole | Otitis | R: 4-8 mg/kg/qd | PO | 10 |
|  |  | 40 | M: 2.8 ± 2.1 yr | 17 (42.5) | Trimethoprim/  sulfamethoxazole | Otitis | R: 4-8 mg/kg/qd | PO | 10 |
|  |  | 40 | M: 3.1 ± 2.5 yr | 18 (45) | Amoxicillin | Otitis | 50 mg/kg/qd | PO | 10 |
| Kaleida PH, et al. (1987) | RCT | 69 | M: 38.5 mo;  N: 32 mo;  R: 5 mo-15 yr | 37 (53.6) | Cefaclor | Otitis | 40 mg/kg/qd | PO | 10 |
|  |  | 64 | M: 46,9 mo;  N: 40 mo;  R: 5 mo-11 yr | 32 (50) | Amoxicillin/  clavulanic acid | Otitis | 40 mg/kg/qd | PO | 10 |
| Risser WL, et al. (1987) | RCT | 109 | M: 7 yr;  R: 6 mo-13 yr | 61 (56) | Cefixime | Cystitis, pharyngitis, pneumonia | 8 mg/kg/qd | IV | R: 10-14 |
| Grubbauer HM, et al. (1990) | Prospective observational | 33 | N: 23 mo;  R: 5 mo-15 yr | NA | Ceftriaxone | Bacterial meningitis | 100 mg/kg/qd | IV | N: 13;  R: 6-23 |
| Skarda DE, et al. (2014) | Clinical trial | 154 | M: 8.5 ± 4.2 yr | 67 (44) | Cefoxitin, piperacillin/  tazobactam | Ruptured appendicitis | 40 mg/kg [cefoxitin], 100 mg/kg/tid [piperacillin/  tazobactam] | IV | 1 [cefoxitin], <4 [piperacillin/  tazobactam] |
| Bradley JS, et al. (2020) | RCT | 74 | R: 1-17 yr | NA | Daptomycin | Acute Hematogenous Osteomyelitis | R: 7-12 mg/kg/qd | IV, PO | R: 14-42 |

**Legend**

bid: twice a day; IM: intramuscular; IQR: interquartile range; IV: intravenous; M: mean; mo: months; N: median; NA: not available; PO: orally; RCT: randomized controlled study; qd: once a day; qid: four times a day;qod: every other day;R: range; UTI: urinary tract infection; yr: years

**References**

1. Ahonkhai, V. I., Cyhan, G. M., Wilson, S. E., and Brown, K. R. (1989). Imipenem-cilastatin in pediatric patients: an overview of safety and efficacy in  studies conducted in the United States. *Pediatr Infect Dis J* 8, 740–744.
2. Al-Fadley, F. (1992). Neutropenia associated with cloxacillin and other semisynthetic penicillins in  children. *Ann Saudi Med* 12, 157–160. doi: 10.5144/0256-4947.1992.157.
3. Ardati, K. O., Thirumoorthi, M. C., and Dajani, A. S. (1979). Intravenous trimethoprim-sulfamethoxazole in the treatment of serious infections in  children. *J Pediatr* 95, 801–806. doi: 10.1016/s0022-3476(79)80740-4.
4. Arguedas, A., Loaiza, C., Herrera, M., and Mohs, E. (1996). Comparative trial of 3-day azithromycin versus 10-day amoxycillin/clavulanate  potassium in the treatment of children with acute otitis media with effusion. *Int J Antimicrob Agents* 6, 233–238. doi: 10.1016/0924-8579(95)00066-6.
5. Arguedas, A., Loaiza, C., Perez, A., Gutierrez, A., Herrera, M. L., and Rothermel, C. D. (2003). A pilot study of single-dose azithromycin versus three-day azithromycin or  single-dose ceftriaxone for uncomplicated acute otitis media in children. *Curr Ther Res Clin Exp* 64, 16–29. doi: 10.1016/j.curtheres.2003.09.005.
6. Arguedas, A., Loaiza, C., Rodriguez, F., Herrera, M. L., and Mohs, E. (1997). Comparative trial of 3 days of azithromycin versus 10 days of clarithromycin in the  treatment of children with acute otitis media with effusion. *J Chemother* 9, 44–50. doi: 10.1179/joc.1997.9.1.44.
7. Asmar, B. I., Maqbool, S., and Dajani, A. S. (1981). Hematologic abnormalities after oral trimethoprim-sulfamethoxazole therapy in  children. *Am J Dis Child* 135, 1100–1103. doi: 10.1001/archpedi.1981.02130360008004.
8. Bégué, P., and Astruc, J. (1995). The overall safety of oral roxithromycin in paediatric clinical studies. *Infection* 23 Suppl 1, S25-7. doi: 10.1007/BF02464956.
9. Bradley, J. S., Arrieta, A. C., Digtyar, V. A., Popejoy, M. W., Grandhi, A., Bokesch, P., et al. (2020). Daptomycin for Pediatric Gram-Positive Acute Hematogenous Osteomyelitis. *Pediatr Infect Dis J* 39, 814–823. doi: 10.1097/INF.0000000000002790.
10. Chonmaitree, T., Congeni, B. L., Munoz, J., Rakusan, T. A., Powell, K. R., and Box, Q. T. (1984). Twice daily ceftriaxone therapy for serious bacterial infections in children. *J Antimicrob Chemother* 13, 511–516. doi: 10.1093/jac/13.5.511.
11. Chu, J. Y., O’Connor, D. M., and Schmidt, R. R. (1977). The mechanism of oxacillin-induced neutropenia. *J Pediatr* 90, 668–669. doi: 10.1016/s0022-3476(77)80413-7.
12. Dagan, R., Syrogiannopoulos, G., Ashkenazi, S., Engelhard, D., Einhorn, M., Gatzola-Karavelli, M., et al. (1994). Parenteral-oral switch in the management of paediatric pneumonia. *Drugs* 47 Suppl 3, 43–51. doi: 10.2165/00003495-199400473-00008.
13. Dubs, M. M. (1985). Beta-lactam-induced neutropenia. *Pediatr Infect Dis* 4, 705–706. doi: 10.1097/00006454-198511000-00037.
14. Dutro, M. P., Piecoro, J. J. J., and Wilson, H. D. (1981). Nafcillin-induced neutropenia in two children. *Am J Hosp Pharm* 38, 889–892.
15. Feldman, S., Doolittle, M., Lott, L., Roberson, P., and Hughes, W. T. (1985). Similar hematologic changes in children receiving trimethoprim-sulfamethoxazole or  amoxicillin for otitis media. *J Pediatr* 106, 995–1000. doi: 10.1016/s0022-3476(85)80257-2.
16. Feldman, W. E., Moffitt, S., and Sprow, N. (1980). Clinical and pharmacokinetic evaluation of parental cefoxitin in infants and  children. *Antimicrob Agents Chemother* 17, 669–674. doi: 10.1128/AAC.17.4.669.
17. Fernando, M., Kankananarachchi, I., Navabalasooriyar, P., Herath, B., and Punchihewa, P. (2019). A Case of Dapsone-Induced Severe Agranulocytosis Causing Life-Threatening Skin  Sepsis in a Sri Lankan Child with Borderline Leprosy: A Success Story! *Case Rep Med* 2019, 2314379. doi: 10.1155/2019/2314379.
18. Greene, G. R., and Cohen, E. (1978). Nafcillin-induced neutropenia in children. *Pediatrics* 61, 94–97.
19. Grubbauer, H. M., Dornbusch, H. J., Dittrich, P., Weippl, G., Mutz, I., Zobel, G., et al. (1990). Ceftriaxone monotherapy for bacterial meningitis in children. *Chemotherapy* 36, 441–447. doi: 10.1159/000238802.
20. Hettmer, S., and Heeney, M. M. (2008). Cefepime-induced neutropenia in a teenager. *Pediatr Blood Cancer* 51, 715–716. doi: 10.1002/pbc.21664.
21. Higham, M., Cunningham, F. M., and Teele, D. W. (1985). Ceftriaxone administered once or twice a day for treatment of bacterial infections  of childhood. *Pediatr Infect Dis* 4, 22–26. doi: 10.1097/00006454-198501000-00007.
22. Hori, C., Hiraoka, M., Tsukahara, H., Tsuchida, S., and Sudo, M. (1997). Intermittent trimethoprim-sulfamethoxazole in children with vesicoureteral reflux. *Pediatr Nephrol* 11, 328–330. doi: 10.1007/s004670050287.
23. Jacobs, R. F., Maples, H. D., Aranda, J. v, Espinoza, G. M., Knirsch, C., Chandra, R., et al. (2005). Pharmacokinetics of intravenously administered azithromycin in pediatric patients. *Pediatr Infect Dis J* 24, 34–39. doi: 10.1097/01.inf.0000148927.48680.fc.
24. Jarkowski, T. L., and Martmer, E. E. (1962). Fatal reaction to sulfadimethoxine (Madribon). A case showing toxic epidermal  necrolysis and leukopenia. *Am J Dis Child* 104, 669–674. doi: 10.1001/archpedi.1962.02080030669012.
25. Kaleida, P. H., Bluestone, C. D., Rockette, H. E., Bass, L. W., Wolfson, J. H., Breck, J. M., et al. (1987). Amoxicillin-clavulanate potassium compared with cefaclor for acute otitis media in  infants and children. *Pediatr Infect Dis J* 6, 265–271. doi: 10.1097/00006454-198703000-00013.
26. Kaplan, S. L., Mason, E. O. J., Kvernland, S. J., Loiselle, E. M., and Feigin, R. D. (1983). Moxalactam treatment of serious infections primarily due to Haemophilus influenzae  type b in children. *Pediatrics* 71, 187–191.
27. Kaplan, S. L., Patterson, L., Edwards, K. M., Azimi, P. H., Bradley, J. S., Blumer, J. L., et al. (2001). Linezolid for the treatment of community-acquired pneumonia in hospitalized  children. Linezolid Pediatric Pneumonia Study Group. *Pediatr Infect Dis J* 20, 488–494. doi: 10.1097/00006454-200105000-00004.
28. Keyserling, H., Feldman, W. E., Moffitt, S., Manning, N., and Hollins, M. (1982). Clinical and pharmacokinetic evaluation of parenteral moxalactam in infants and  children. *Antimicrob Agents Chemother* 21, 898–901. doi: 10.1128/AAC.21.6.898.
29. Kitzing, W., Nelson, J. D., and Mohs, E. (1981). Comparative toxicities of methicillin and nafcillin. *Am J Dis Child* 135, 52–55. doi: 10.1001/archpedi.1981.02130250040013.
30. Kumar, K., and Kumar, A. (1981). Reversible neutropenia associated with ampicillin therapy in pediatric patients. *Drug Intell Clin Pharm* 15, 802–806. doi: 10.1177/106002808101501015.
31. Leventhal, J. M., and Silken, A. B. (1976). Oxacillin-induced neutropenia in children. *J Pediatr* 89, 769–771. doi: 10.1016/s0022-3476(76)80800-1.
32. Losurdo, G., Castagnola, E., Cristina, E., Tasso, L., Toma, P., Buffa, P., et al. (1998). Cervical lymphadenitis caused by nontuberculous mycobacteria in immunocompetent  children: clinical and therapeutic experience. *Head Neck* 20, 245–249. doi: 10.1002/(sici)1097-0347(199805)20:3<245::aid-hed10>3.0.co;2-j.
33. Patel, S., Burzio, V., Green, H., Rees, S., Tebruegge, M., Jones, C., et al. (2018). The Impact of Pediatric Outpatient Parenteral Antibiotic Therapy Implementation at a  Tertiary Children’s Hospital in the United Kingdom. *Pediatr Infect Dis J* 37, e292–e297. doi: 10.1097/INF.0000000000002031.
34. Pietroni, M. (2005). Flucloxacillin associated neutropenia in children treated for bone and joint  infections (J. Paediatr. Child Health 2005; 41: 48-51). *J Paediatr Child Health* 41, 534. doi: 10.1111/j.1440-1754.2005.00700_1.x.
35. Principi, N., Marchisio, P., Biasini, A., Dalla Villa, A., and Biasini, G. (1984). Early and late neutropenia in children treated with cotrimoxazole  (trimethoprim-sulfamethoxazole). *Acta Paediatr Scand* 73, 763–767. doi: 10.1111/j.1651-2227.1984.tb17772.x.
36. Risser, W. L., Barone, J. S., Clark, P. A., and Simpkins, D. L. (1987). Noncomparative, open label, multicenter trial of cefixime for treatment of bacterial  pharyngitis, cystitis and pneumonia in pediatric patients. *Pediatr Infect Dis J* 6, 1002–1006. doi: 10.1097/00006454-198710000-00043.
37. Schaad, U. B., Pfenninger, J., and Wedgewood-Krucko, J. (1987). Sequential intravenous-oral amoxycillin/clavulanate (Augmentin) therapy in  paediatric hospital practice. *J Antimicrob Chemother* 19, 385–391. doi: 10.1093/jac/19.3.385.
38. Shinohara, Y. T., and Colbert, J. (1994). Vancomycin-induced neutropenia during treatment of endocarditis in a pediatric  patient. *Ann Pharmacother* 28, 723–726. doi: 10.1177/106002809402800607.
39. Skarda, D. E., Schall, K., Rollins, M., Andrews, S., Olson, J., Greene, T., et al. (2014). Response-based therapy for ruptured appendicitis reduces resource utilization. *J Pediatr Surg* 49, 1726–1729. doi: 10.1016/j.jpedsurg.2014.09.012.
40. St John, M. A., and Prober, C. G. (1981). Side effects of cloxacillin in infants and children. *Can Med Assoc J* 125, 458–460.
41. Tuomanen, E. I., Powell, K. R., Marks, M. I., Laferriere, C. I., Altmiller, D. H., Sack, C. M., et al. (1981). Oral chloramphenicol in the treatment of Haemophilus influenzae meningitis. *J Pediatr* 99, 968–974. doi: 10.1016/s0022-3476(81)80035-2.
42. van den Boom, J., Kristiansen, J. B., Voss, L. M., and Stott, N. S. (2005). Flucloxacillin associated neutropenia in children treated for bone and joint  infections. *J Paediatr Child Health* 41, 48–51. doi: 10.1111/j.1440-1754.2005.00535.x.
43. Wee, I. Y., and Oh, H. M. (2001). Teicoplanin-induced neutropenia in a paediatric patient with vertebral  osteomyelitis. *Scand J Infect Dis* 33, 157–158. doi: 10.1080/003655401750065599.
44. Yusef, D., Gonzalez, B. E., Foster, C. B., Goldfarb, J., Saracusa, C., Worley, S., et al. (2017). Piperacillin-Tazobactam-induced Adverse Drug Events in Pediatric Patients on Outpatient Parenteral Antimicrobial Therapy. *Pediatr Infect Dis J* 36, 50–52. doi: 10.1097/INF.0000000000001351.
