# Supplementary material for: Antibiotic-Induced Neutropenia in Pediatric Patients: New Insights From Pharmacoepidemiological Analyses and a Systematic Review
Source: Front Pharmacol. 2022 Jun 2;13:877932. doi: 10.3389/fphar.2022.877932 (PMC9201445; doi:10.3389/fphar.2022.877932)
Supplement: Supplementary file 4 [file Table4.DOCX]

**Supplementary Table 4.** Details of neutropenia occurrence in the studies excluded from TTO analysis.

| **Main author (year)** | **Antibiotic**  **[ATC Code]** | **N patients with neutropenia**  **(% out of total patients)** | **N neutrophils/mmc** | **TTO**  **(days)** | **Antibiotic withdrawal** | **Resolution (days)** | **Symptoms or complications** | **Other associated ADR** |
| --- | --- | --- | --- | --- | --- | --- | --- | --- |
| Kitzing W, et al. (1981) | Methicillin  [J01CF03] | 6 (24) | <1,500 | NA | Y | NA | NA | Eosinophilia, elevated liver enzymes |
|  | Nafcillin  [J01CF06] | 5 (20) | <1,500 | NA | Y | NA | Death (1 pt) | NA |
| Keyserling H, et al. (1982) | Moxalactam  [J01DD06] | 5 (14) | <1,500 | NA | N | NA | NA | NA |
| Principi N, et al. (1984) | Trimethoprim/  sulfamethoxazole  [J01EE01] | 14 (35) | <1,500 | NA | N | 7-14 | N | Eosinophilia |
|  | Trimethoprim/  sulfamethoxazole  [J01EE01] | 7 (17.5) | <1,500 | NA | N | NA | N | Eosinophilia |
|  | Amoxicillin  [J01CA04] | 6 (13.3) | <1,500 | NA | N | NA | N | Eosinophilia |
| Kaleida PH, et al. (1987) | Cefaclor  [J01DC04] | 1 (1.4) | <1,500 | NA | N | NA | N | Eosinophilia |
|  | Amoxicillin/  clavulanic acid  [J01CR02] | 6 (9.3) | <1,500 | NA | N | NA | N | NA |
| Risser WL, et al. (1987) | Cefixime  [J01DD08] | 1 | 950 | NA | NA | NA | N | Diarrhea, vomit, rash |
| Grubbauer HM, et al. (1990) | Ceftriaxone  [J01DD04] | 1 (3) | <1,500 | NA | NA | NA | NA | NA |
| Skarda DE, et al. (2014) | Cefoxitin, piperacillin/  tazobactam  [J01DC01, J01CR05] | 4 (3) | NA | NA | NA | NA | NA | NA |
| Bradley JS, et al. (2020) | Daptomycin  [J01XX09] | 1 (1) | NA | NA | NA | NA | NA | NA |

**Legend:** M: mean; N: no; NA: not available; pt: patients; R: range; Y: yes
