# Supplementary material for: Antibiotic-Induced Neutropenia in Pediatric Patients: New Insights From Pharmacoepidemiological Analyses and a Systematic Review
Source: Front Pharmacol. 2022 Jun 2;13:877932. doi: 10.3389/fphar.2022.877932 (PMC9201445; doi:10.3389/fphar.2022.877932)
Supplement: Supplementary file 5 [file DataSheet1.docx]

**Supplementary Material. Full search strategy for Pubmed.**

(“anti bacterial agent”[TIAB] OR “anti infective agents”[TIAB] OR antibacterial[TIAB] OR “anti-bacterial agents”[TIAB] OR antibiotic*[TIAB] OR “antiinfective agent”[TIAB] OR antimicrobial[TIAB] OR antiseptic[TIAB] OR chemotherapeutical [TIAB] OR “microbiological agent”[TIAB] OR aminoglycoside*[TIAB] OR carbapenem[TIAB] OR cefalosporine[TIAB] OR cephalosporin[TIAB] OR fluoroquinolone[TIAB] OR "glycopeptide antibiotic"[TIAB] OR "glycopeptide antibiotics"[TIAB] OR lipoglycopeptide*[TIAB] OR macrolide*[TIAB] OR macrotetrolide[TIAB] OR monobactam*[TIAB] OR oxazolidinone[TIAB] OR penicillin*[TIAB] OR "polypeptide antibiotic"[TIAB] OR "polypeptide antibiotics"[TIAB] OR rifampicin[TIAB] OR sulfamide[TIAB] OR streptogramin*[TIAB] OR tetracylin*[TIAB] OR "beta lactam antibiotic"[TIAB] OR "beta lactam antibiotics"[TIAB] OR vancomycin[TIAB] OR teicoplanin[TIAB] OR telavancin[TIAB] OR dalbavancin[TIAB] OR oritavancin[TIAB] OR “polymyxin B”[TIAB] OR “fusidic acid”[TIAB] OR metronidazole[TIAB] OR tinidazole[TIAB] OR ornidazole[TIAB] OR nitrofurantoin[TIAB] OR nifurtoinol[TIAB] OR furazidin[TIAB] OR fosfomycin[TIAB] OR xibornol[TIAB] OR clofoctol[TIAB] OR spectinomycin[TIAB] OR methenamine[TIAB] OR “mandelic acid”[TIAB] OR nitroxoline[TIAB] OR linezolid[TIAB] OR daptomycin[TIAB] OR bacitracin[TIAB] OR tedizolid[TIAB] OR lefamulin[TIAB] OR ofloxacin[TIAB] OR ciprofloxacin[TIAB] OR pefloxacin[TIAB] OR enoxacin[TIAB] OR temafloxacin[TIAB] OR norfloxacin[TIAB] OR lomefloxacin[TIAB] OR fleroxacin[TIAB] OR sparfloxacin[TIAB] OR rufloxacin[TIAB] OR grepafloxacin[TIAB] OR levofloxacin[TIAB] OR trovafloxacin[TIAB] OR moxifloxacin[TIAB] OR gemifloxacin[TIAB] OR gatifloxacin[TIAB] OR prulifloxacin[TIAB] OR pazufloxacin[TIAB] OR garenoxacin[TIAB] OR sitafloxacin[TIAB] OR tosufloxacin[TIAB] OR delafloxacin[TIAB] OR levonadifloxacin[TIAB] OR lascufloxacin[TIAB] OR rosoxacin[TIAB] OR “nalidixic acid”[TIAB] OR “piromidic acid”[TIAB] OR “pipemidic acid”[TIAB] OR “oxolinic acid”[TIAB] OR cinoxacin[TIAB] OR flumequine[TIAB] OR nemonoxacin[TIAB] OR streptomycin[TIAB] OR streptoduocin[TIAB] OR tobramycin[TIAB] OR gentamicin[TIAB] OR kanamycin[TIAB] OR neomycin[TIAB] OR amikacin[TIAB] OR netilmicin[TIAB] OR sisomicin[TIAB] OR dibekacin[TIAB] OR ribostamycin[TIAB] OR isepamicin[TIAB] OR arbekacin[TIAB] OR bekanamycin[TIAB] OR plazomicin[TIAB] OR erythromycin[TIAB] OR spiramycin[TIAB] OR midecamycin[TIAB] OR oleandomycin[TIAB] OR roxithromycin[TIAB] OR josamycin[TIAB] OR troleandomycin[TIAB] OR clarithromycin[TIAB] OR azithromycin[TIAB] OR miocamycin[TIAB] OR rokitamycin[TIAB] OR dirithromycin[TIAB] OR flurithromycin[TIAB] OR telithromycin[TIAB] OR solithromycin[TIAB] OR clindamycin[TIAB] OR lincomycin[TIAB] OR pristinamycin[TIAB] OR “quinupristin/dalfopristin”[TIAB] OR trimethoprim[TIAB] OR brodimoprim[TIAB] OR iclaprim[TIAB] OR sulfaisodimidine[TIAB] OR sulfamethizole[TIAB] OR sulfadimidine[TIAB] OR sulfapyridine[TIAB] OR sulfafurazole[TIAB] OR sulfanilamide[TIAB] OR sulfathiazole[TIAB] OR sulfathiourea[TIAB] OR sulfamethoxazole[TIAB] OR sulfadiazine[TIAB] OR sulfamoxole[TIAB] OR sulfadimethoxine[TIAB] OR sulfalene[TIAB] OR sulfametomidine[TIAB] OR sulfametoxydiazine[TIAB] OR sulfamethoxypyridazine[TIAB] OR sulfaperin[TIAB] OR sulfamerazine[TIAB] OR sulfaphenazole[TIAB] OR sulfamazone[TIAB] OR “ceftobiprole medocaril”[TIAB] OR “ceftaroline fosamil”[TIAB] OR faropenem[TIAB] OR cefiderocol[TIAB] OR meropenem[TIAB] OR ertapenem[TIAB] OR doripenem[TIAB] OR biapenem[TIAB] OR “tebipenem pivoxil”[TIAB] OR aztreonam[TIAB] OR carumonam[TIAB] OR cefepime[TIAB] OR cefpirome[TIAB] OR cefozopran[TIAB] OR demeclocycline[TIAB] OR doxycycline[TIAB] OR chlortetracycline[TIAB] OR lymecycline[TIAB] OR metacycline[TIAB] OR oxytetracycline[TIAB] OR minocycline[TIAB] OR rolitetracycline[TIAB] OR penimepicycline[TIAB] OR clomocycline[TIAB] OR tigecycline[TIAB] OR eravacycline[TIAB] OR sarecycline[TIAB] OR omadacycline[TIAB] OR chloramphenicol[TIAB] OR thiamphenicol[TIAB] OR ampicillin[TIAB] OR pivampicillin[TIAB] OR carbenicillin[TIAB] OR amoxicillin[TIAB] OR carindacillin[TIAB] OR bacampicillin[TIAB] OR epicillin[TIAB] OR pivmecillinam[TIAB] OR azlocillin[TIAB] OR mezlocillin[TIAB] OR mecillinam[TIAB] OR piperacillin[TIAB] OR ticarcillin[TIAB] OR metampicillin[TIAB] OR talampicillin[TIAB] OR sulbenicillin[TIAB] OR temocillin[TIAB] OR hetacillin[TIAB] OR aspoxicillin[TIAB] OR benzylpenicillin[TIAB] OR phenoxymethylpenicillin[TIAB] OR propicillin[TIAB] OR azidocillin[TIAB] OR pheneticillin[TIAB] OR penamecillin[TIAB] OR clometocillin[TIAB] OR benzathine[TIAB] OR procaine[TIAB] OR dicloxacillin[TIAB] OR cloxacillin[TIAB] OR meticillin[TIAB] OR oxacillin[TIAB] OR flucloxacillin[TIAB] OR nafcillin[TIAB] OR sulbactam[TIAB] OR tazobactam[TIAB] OR sultamicillin[TIAB] OR cefalexin[TIAB] OR cefaloridine[TIAB] OR cefalotin[TIAB] OR cefazolin[TIAB] OR cefadroxil[TIAB] OR cefazedone[TIAB] OR cefatrizine[TIAB] OR cefapirin[TIAB] OR cefradine[TIAB] OR cefacetrile[TIAB] OR cefroxadine[TIAB] OR ceftezole[TIAB] OR cefoxitin[TIAB] OR cefuroxime[TIAB] OR cefamandole[TIAB] OR cefaclor[TIAB] OR cefotetan[TIAB] OR cefonicid[TIAB] OR cefotiam[TIAB] OR loracarbef[TIAB] OR cefmetazole[TIAB] OR cefprozil[TIAB] OR ceforanide[TIAB] OR cefminox[TIAB] OR cefbuperazone[TIAB] OR flomoxef[TIAB] OR cefotaxime[TIAB] OR ceftazidime[TIAB] OR cefsulodin[TIAB] OR ceftriaxone[TIAB] OR cefmenoxime[TIAB] OR latamoxef[TIAB] OR ceftizoxime[TIAB] OR cefixime[TIAB] OR cefodizime[TIAB] OR cefetamet[TIAB] OR cefpiramide[TIAB] OR cefoperazone[TIAB] OR cefpodoxime[TIAB] OR ceftibuten[TIAB] OR cefdinir[TIAB] OR cefditoren[TIAB] OR cefcapene[TIAB] OR cefteram[TIAB] OR tyrothricin[TIAB] OR fusafungine[TIAB] OR gramicidin[TIAB] OR nystatin[TIAB] OR natamycin[TIAB] OR paromomycin[TIAB] OR “amphotericin B”[TIAB] OR kanamycin[TIAB] OR colistin[TIAB] OR rifaximin[TIAB] OR fidaxomicin[TIAB] OR rifamycin[TIAB]) AND (child*[TIAB] OR adolescent[TIAB] OR infant[TIAB] OR paediatric*[TIAB] OR pediatric*[TIAB] OR teenager[TIAB]) AND (neutropaenia[TIAB] OR neutropenia[TIAB] OR pancytopaenia[TIAB] OR pancytopenia[TIAB] OR panhemocytopaenia[TIAB] OR panhemocytopenia[TIAB] OR neutrophil*[TIAB] “blood cell count”[TIAB] OR “blood count”[TIAB] OR “blood cell”[TIAB] OR “blood cells”[TIAB] OR “hematocyte”[TIAB] OR “hemocyte”[TIAB] OR “hemocytes”[TIAB]OR “leucocyte depletion”[TIAB] OR leucocytopaenia[TIAB] OR leucocytopenia[TIAB] OR leucopaenia[TIAB] OR leucopenia[TIAB] OR “leukocyte depletion”[TIAB] OR leukocytopenia[TIAB] OR leukopaenia[TIAB] OR leukopenia[TIAB] OR safety[TIAB] OR “adverse effect”[TIAB] OR “adverse effects”[TIAB] OR “adverse event”[TIAB] OR “adverse events”[TIAB] OR “adverse reaction”[TIAB] OR “adverse drug”[TIAB] OR “side effect”[TIAB] OR “side effects”[TIAB])
